# Epigenetic control of the ferric uptake regulator (Fur) and fumarate nitrate reductase (FNR) master regulatory proteins contributes to *Haemophilus influenzae* survival during lung infection

**DOI:** 10.1128/mbio.01355-25

**Published:** 2025-07-23

**Authors:** Celia Gil-Campillo, Begoña Euba, Irene Rodríguez-Arce, David San León, Mary C. Marino, Javier Asensio-López, Nahikari López-López, Joshua C. Mell, Gabriel Gutiérrez, Jeroen D. Langereis, María Antonia Sánchez-Romero, Junkal Garmendia

**Affiliations:** 1 Instituto de Agrobiotecnología, Consejo Superior de Investigaciones Científicas (IdAB-CSIC)-Gobierno de Navarra, Mutilva, Spain; 2 Centro de Investigación Biomédica en Red de Enfermedades Respiratorias (CIBERES) 568067, Madrid, Spain; 3 Department of Systems Biology, Centro Nacional de Biotecnología 54447, Madrid, Spain; 4 Interdisciplinary Platform for Sustainable Plastics towards a Circular Economy-Spanish National Research Council (SusPlast-CSIC), Madrid, Spain; 5 Department of Microbiology and Immunology, College of Medicine, Drexel University 12312, Philadelphia, Pennsylvania, USA; 6 Center for Genomic Sciences, Institute of Molecular Medicine and Infectious Disease, Drexel University 6527, Philadelphia, Pennsylvania, USA; 7 Asociación de la Industria Navarra (AIN)-Gobierno de Navarra, Cordovilla, Spain; 8 Departamento de Genética, Facultad de Biología, Universidad de Sevilla 16778, Seville, Andalucía, Spain; 9 Department of Laboratory Medicine, Laboratory of Medical Immunology, Radboud Community for Infectious Diseases, Radboud University Medical Center 6034, Nijmegen, the Netherlands; 10 Departamento de Microbiología y Parasitología, Facultad de Farmacia, Universidad de Sevilla 16778, Seville, Andalucía, Spain; 11 Conexión Antimicrobial Resistance-Spanish National Research Councial (AMR-CSIC), Madrid, Spain; University of Minnesota Twin Cities, Minneapolis, Minnesota, USA

**Keywords:** *Haemophilus influenzae*, *in vivo* Tn-seq, airway infection, epigenetic regulation of gene expression, DNA adenine Dam methylation, FNR regulon, Fur regulation, gene expression heterogeneity

## Abstract

DNA regulatory elements that dictate how the bacterial pathobiont *Haemophilus influenzae* infects and adapts to the airways of immunocompromised patients suffering from chronic obstructive pulmonary disease (COPD) are poorly understood. This is in part due to the scarcity of research integrating genetic and epigenetic perspectives to shed light on the role of distinct bacterial adaptive strategies within the human airways. In this work, global fitness profiling of *H. influenzae* mutants by high-throughput transposon mutant sequencing within the mouse lung identified Dam methyltransferase as an *in vivo* requirement for respiratory infection. Equally, single-molecule real-time sequencing methylome analyses found undermethylation of GATC motifs within putative regulatory elements and revealed the first case of phenotypic variation controlled by variable Dam methylation in *H. influenzae*. Moreover, RNA sequencing differential gene expression disclosed a novel regulatory network where Dam methyltransferase positively regulates the expression of the ferric uptake regulator (Fur), which in turn represses the expression of the fumarate nitrate reductase (FNR) regulator and, subsequently, of a repertoire of genes that belong to the FNR regulon and encode bacterial anaerobic defenses against, among others, reactive nitrogen species produced within the diseased airways. Our results present a multifactorial regulatory network where the interplay between the Fur and FNR master transcriptional regulators is controlled epigenetically by Dam methylation. We put forward the notion that this network regulates *H. influenzae* survival in diseased airway niches with high nitrosative stress where damage reduces the amount of oxygen in the lungs, as encountered in COPD.

**IMPORTANCE:**

Regulatory mechanisms governing the ability of *Haemophilus influenzae* to survive within the human lungs remain poorly elucidated. Here, by coordinated exploitation of multiomic approaches, and using reference and clinical strains, we present evidence that the Dam methyltransferase mediates epigenetic regulatory mechanisms facilitating bacterial phenotypic diversity and flexibility, besides reversibility, to contribute to *H. influenzae* survival within the lungs of individuals where disease reduces the amount of oxygen, as encountered in COPD. We reveal a novel bacterial network where DNA methylation regulates the expression of and interplay between the Fur and FNR master transcriptional regulators, which act in a coordinated manner, controlling the expression of *H. influenzae* genes involved in bacterial defenses against the nitrosative stress encountered in the diseased lungs, and further highlight the importance of oxygen restriction within this hostile niche.

## INTRODUCTION

Chronic obstructive pulmonary disease (COPD) is a recognizable pattern of chronic symptoms and structural and functional impairments of the lung that arise due to the accumulation of gene–environment interactions faced by an individual over the lifespan (1, 2). Enhanced levels of reactive oxygen species (ROS) and reactive nitrogen species (RNS) leading to high nitrosative stress are a hallmark of severe COPD, which amplifies inflammatory processes in the lung parenchyma, thereby causing cell damage (3). Microbial dysbiosis is present in COPD, where a reduction in the diversity of lung bacterial composition has been linked to disease progression. Airway bacterial infections complicate the disease course in most COPD patients, leading to increased symptoms, faster decline in lung function, acute exacerbations, and reduced quality of life (4). Within this dysbiotic niche, damage arising from COPD fosters alveolar hypoxia, leading to low-oxygen microenvironments that modulate the host–pathogen interplay in the diseased lung tissue (5). Deciphering bacterial regulatory elements that influence lung pathogenic infection will inform additional therapeutic interventions.

The highly diverse human-restricted species *Haemophilus influenzae* is a normal part of the upper airway microbiome that causes infections in susceptible hosts. Infant mortality due to invasive infections was dramatically reduced by the introduction of the conjugated vaccine that targets strains with the serotype *b* polysaccharide capsule (6). However, non-typeable *H. influenzae* strains continue to cause high morbidity due to their role in common infections and chronic diseases and are major contributors to persistent infection and exacerbations of COPD (7–10).

DNA regulatory elements that dictate how *H. influenzae* infects the airways are poorly understood, in part due to the lack of research integrating genetic and epigenetic perspectives to pursue bacterial adaptive strategies within the diseased lungs. From a genetic perspective, the *H. influenzae* anoxic redox control ArcA/ArcB two-component signal transduction system is active under low-oxygen conditions and functions via the sensor kinase ArcB to activate the DNA binding response regulator ArcA. Phosphorylated ArcA controls a set of genes involved in adaptation to respiratory conditions of growth, and mutants lacking ArcA are attenuated for survival in murine models of pathogenesis, suggesting that *H. influenzae* encounters low-oxygen microenvironments within the host (11). *H. influenzae* also uses the fumarate nitrate reductase (FNR) global regulator to regulate gene expression for anaerobic defense against exposure to nitric oxide (NO) donors and interferon-γ-treated macrophages (12). Careful regulation of iron and iron-containing moieties uptake via the ferric uptake regulator (Fur) is also essential for *H. influenzae* homeostasis and disease progression, as it lacks the ability to synthesize heme (13). Although unknown for *H. influenzae*, Fur is controlled by O_2_ availability in *Escherichia coli* as labile Fe^2+^ pool is higher under anaerobic conditions, driving the formation of more Fe^2+^-Fur and, accordingly, more DNA binding (14).

Conversely, *H. influenzae* uses the OxyR system to prevent damage by ROS as it detects reactive oxygen, is activated by peroxide oxidation, and coordinates the expression of defensive antioxidants (15). Indeed, OxyR regulation of the *gtr* and *opvAB* operons in *Salmonella enterica* provides well-known examples of bistable gene expression also involving epigenetic regulatory mechanisms by the means of Dam DNA methylation-dependent switches (16). The Dam methyltransferase methylates the adenosine moiety in 5′-GATC-3′ motifs. In *E. coli* and *S. enterica*, most GATC sites are methylated on both strands, except for transient hemimethylation after passage of the DNA replication fork. However, particular GATC sites remain stably undermethylated when Dam activity is prevented by protein binding to DNA regions containing Dam motifs (17). The combination of methylated and undermethylated GATC sites at promoters and regulatory regions may indicate a form of transcriptional epigenetic control and a source of heterogeneity that may give rise to distinct phenotypic lineages, i.e., OFF and ON cells (16–21). In *H. influenzae*, a role for Dam methyltransferase in invasive infection has been reported (22). However, *H. influenzae* genome-wide Dam GATC methylation, its contribution to bacterial gene expression control, and a possible relationship with regulatory proteins accounting for genetic and epigenetic regulation within the lungs are unexplored aspects.

In this work, we used a mouse lung infection model and transposon mutant sequencing (Tn-seq) to screen *in vivo* the relative fitness of a library containing thousands of independent *H. influenzae* transposon insertion mutants. Among others, we identified a key role for the *dam* methyltransferase-encoding gene. To pursue epigenetic regulation of *H. influenzae* gene expression, genome-wide investigation of Dam methylation patterns was performed by single-molecule real-time (SMRT) sequencing-based DNA methylome analyses, and genome-wide changes in gene expression were evaluated using RNA sequencing (RNA-seq). These multiomic approaches, followed by detailed mechanistic analysis of specific genes, uncovered *H. influenzae* phenotypic variation controlled by Dam methylation and a novel multifactorial regulatory network where Dam methylation controls the interplay between the Fur and FNR global transcriptional regulators, in such a way that Fur is a repressor of *fnr* expression. These novel regulatory mechanisms aim to control the expression of bacterial defenses in a coordinated manner and may provide an adaptive benefit for *H. influenzae* in limiting oxygen environments such as those found in the lungs of COPD patients.

## MATERIALS AND METHODS

### Bacterial strains and media

Strains used in this study are listed in Table S1. *H. influenzae* clinical strains were recovered from COPD respiratory samples (BioProject PRJNA282520) (23). *H. influenzae* strains were grown at 37°C on PolyViteX (PVX) agar (bioMérieux, 43101), on brain-heart infusion agar (Condalab, 1400.10) supplemented with 10 µg/mL hemin (Merck, H9039) and 10 µg/mL nicotinamide adenine dinucleotide (Merck, N0632), referred to as supplemented brain heart infusion (sBHI) agar, or on supplemented *Haemophilus* test medium agar (Oxoid, CM0898), referred to as supplemented *Haemophilus* test medium (sHTM) agar. *H. influenzae* liquid cultures were grown at 37°C in sBHI. Solid and liquid cultures were grown aerobically in 5% CO_2_; alternatively, solid and liquid cultures were grown under anaerobic conditions in an anaerobic workstation (Don Withlety A25). In all cases, *H. influenzae* strains were grown on PVX agar for 12 h in aerobiosis or anaerobiosis; depending on the assay, growth was monitored as detailed in the Supplemental Methods. To test *H. influenzae* sensitivity to S-nitrosoglutathione (GSNO; Santa Cruz Biotechnology, sc-200349B), GSNO was used at a final concentration of 0.5 mM (for further details, see the Supplemental Material). Erythromycin 11 µg/mL (Erm_11_) or spectinomycin 50 µg/mL (Spec_50_) was used when required. *Escherichia coli* was grown on Luria–Bertani (LB) agar at 37°C, with ampicillin 100 µg/mL (Amp_100_), Erm 150 µg/mL, or Spec_50_, when necessary.

### Generation of *H. influenzae* mutant strains

Plasmids and primers are shown in Table S2 and S3, respectively. For the generation of *H. influenzae* mutants, a DNA fragment containing each gene/operon and its respective flanking regions was PCR amplified with Phusion polymerase (Fisher Scientific) using RdKW20/P621/P665 genomic DNA as template and primers gene + flanking region-F1 and gene + flanking region-R1, and cloned into pJET1.2/blunt (Fisher Scientific), generating a range of pJET1.2-*gene* plasmids. Four gene disruption strategies were employed:

- Erm-based disruption strategy. In each case, the cloned PCR product was disrupted by inverse PCR with Phusion polymerase using primers *gene*-F2 and *gene*-R2. An internal fragment was replaced by a blunt-ended *ermC* resistance cassette excised by *Sma*I digestion from pBSLerm (24), generating the respective set of pJET1.2-*gene::ermC* plasmids, used as a template to amplify each *gene::ermC* disruption cassette with primers gene + flanking region-F1 and gene + flanking region-R1 (used to generate strains RdKW20Δ*sspA*/P953, RdKW20Δ*atpD*/P1011, RdKW20Δ*znuA*/P1012, RdKW20∆*dam*/P1022, P621∆*dam*/P1023, P665∆*dam*/P1024, RdKW20∆*fnr*/P1220, and RdKW20∆*dam*∆*fnr*/P1231).
- Spec-based disruption strategy A. In each case, the cloned PCR product was disrupted by inverse PCR with Phusion polymerase using primers *gene*-F2 and *gene*-R2. An internal fragment was replaced by a blunt-ended Spec resistance cassette excised by *EcoR*V digestion from pRSM2832 (25), generating the respective collection of pJET1.2-*gene::spec* plasmids, used as a template to amplify each *gene::spec* disruption cassettes with primers gene + flanking region-F1 and gene + flanking region-R1 (used to generate strains RdKW20∆*fnr*/P1221 and RdKW20∆*dam*∆*fnr*/P1231).
- Spec-based disruption strategy B. For each gene, a Spec resistance gene was independently PCR amplified from pRSM2832 using gene-specific mutagenic primers *gene*-F2 and *gene*-R2. *E. coli* SW102 cells were prepared for recombineering, co-electroporated with pJET1.2-*gene* (Amp^r^) (50 ng) and the *gene*-specific mutagenic cassette (Spec^r^) (200 ng) (25). Mutagenized clones containing pJET1.2-*gene::spec* were selected on LB agar with Amp_100_ and Spec_50_. This plasmid was used as a template to amplify the *gene::spec* disruption cassette with primers *gene*-F1 and *gene*-R1 (used to generate strain RdKW20∆*fur*/P583).
- Spec-based disruption strategy C. To make the RdKW20∆*relA* knockout (P1503), a deletion construct was produced by overlap PCR that contained a Spec resistance cassette flanked by regions of ~1 kb up- and downstream of the *relA* gene (HI_0334, encoding GTP diphosphokinase). DreamTaq PCR master mix (Thermo Fisher) was used for PCR reactions. The Spec^R^ cassette was generated from plasmid template pR412 using the *SpecC* primers, and the up- and downstream regions of *relA* were amplified from RdKW20 genomic DNA using *relA_flankA* and *relA_flankB* primers, respectively. The *relA-*proximal primer for each flank contained 50-nt 5´-overhangs identical to the two ends of the Spec^R^ cassette. The flanking fragments were attached to the Spec^R^ cassette using two serial annealing, extension, and PCR reactions.

In all cases, disruption cassettes were independently used to transform *H. influenzae* strains by using the M-IV method (26, 27). Transformants were selected on sHTM agar with Erm_11_ or Spec_50_ to obtain RdKW20∆*dam*, RdKW20Δ*relA*, RdKW20Δ*sspA*, RdKW20Δ*atpD*, RdKW20Δ*znuA*, RdKW20∆*fnr*, RdKW20∆*fur*, RdKW20∆*dam*∆*fnr*, P621∆*dam,* and P665∆*dam* mutants. In all cases, mutations were confirmed by PCR.

### Generation of *H. influenzae htpG::gfp* and *nif3::gfp* fusion strains

First, the pZEP07 plasmid (28) was *EcoR*V digested, dephosphorylated, and ligated to the Spec^R^ cassette previously obtained by *EcoR*V digestion of the pSRM2832 plasmid, generating pZEP07::Spec^R^. Next, a DNA fragment containing each gene, i.e., *htpG* and *nif3*, and their respective downstream regions, was PCR amplified with Phusion polymerase using RdKW20 genomic DNA as template and primers FragA-*gene*-F1 and FragB-*gene*-R1, and cloned into pJET1.2/blunt, generating pJET1.2-*htpG + downstream* and pJET1.2-*nif3 + downstream* plasmids, which were then used as template for inverse PCR, 9 bp downstream of the stop codon of each gene, with Phusion polymerase and primers FragB-*gene*-F1 and FragA-*gene-*R1. Next, a fragment containing the promoterless green fluorescent protein (*gfp*) gene and the Spec^R^ cassette was amplified from pZEP07::Spec^R^ using primers GFP-F1 and Spec_R4, and ligated into the inverse PCR products, generating pJET1.2-*htpG::gfp* and pJET1.2-*nif3::gfp*. These plasmids were used as templates to amplify the *gene::gfp::*Spec^R^ cassettes with primers FragA-*gene*-F1 and FragB-*gene*-R1. Cassettes were independently transformed into *H. influenzae* strains by using the M-IV method. Transformants were selected on sHTM agar with Spec_50_, generating RdKW20WT-*htpG::gfp*, RdKW20∆*dam-htpG::gfp*, RdKW20∆*fnr-htpG::gfp*, RdKW20WT-*nif3::gfp*, RdKW20∆*dam-nif3::gfp*, P621WT-*htpG::gfp,* P621∆*dam-htpG::gfp*, P665WT-*htpG::gfp*, and P665∆*dam-htpG::gfp*. In all cases, mutations were confirmed by PCR.

### Generation of *H. influenzae* strains with modified promoter regions

- For strains with modified *htpG* promoter region, promoter modification cassettes were synthesized by GenScript and provided as pUC18-derivative plasmids pUC18-*htpG-*WT and pUC18-*htpG*-GATC1. A fragment containing the 3′-end of the *nif3* gene, an ErmC^R^ cassette, the wild-type (WT) or modified (GATC to AATC) *htpG* regulatory region, and the *htpG* gene coding sequence were synthesized and cloned with *BamH*I flanking restriction sites. Linear cassettes generated by *BamH*I digestion were independently transformed in the *H. influenzae* RdKW20-*htpG::gfp* strain by using the M-IV method. Transformants were selected on sHTM agar with ErmC_11_, generating RdKW20WT-*htpG-*WT::*gfp* and RdKW20WT-*htpG-*AATC::*gfp* isogenic strains.
- For strains with modified *dmsA* promoter region, promoter modification cassettes were synthesized by GenScript and provided as pUC18-derivative plasmids pUC18-*dmsA-*WT and pUC18-*dmsA-*GCTC. A fragment containing the 3′-end of the HI_1078 gene, a Spec^R^ cassette, the WT or modified (GATC to GCTC) *dmsA* regulatory region, and the *dmsA* gene coding sequence was synthesized and cloned with *BamH*I flanking restriction sites. Linear cassettes generated by *BamH*I digestion were independently transformed into *H. influenzae* strains by using the M-IV method. Transformants were selected on sHTM agar with Spec_50_, generating RdKW20 WT-*dmsA-*WT, RdKW20∆*dam-dmsA-*WT, and RdKW20 WT-*dmsA-*GCTC strains.
- For strains with modified *fnr* promoter region, promoter modification cassettes were synthesized by GenScript and provided as pUC18-derivative plasmids pUC18-*fnr*WT, pUC18-*fnr*GCTC, pUC18-*fnr*BS*, and pUC18-*fnr*-FurBS*. A fragment containing the 3′-end of the HI_1424 gene, a Spec^R^ cassette, the WT or modified (GATC to GCTC; TTGCGTTAGATCAA to GGTATGGAGATCAA; AACATAATTAAAATT to CCACGCCGGCCCCGG) FNR regulatory region, and the *fnr* gene coding sequence were synthesized and cloned with *BamH*I flanking restriction sites. Linear cassettes generated by *BamH*I digestion were independently transformed into *H. influenzae* strains by using the M-IV method. Transformants were selected on sHTM agar with Spec_50_, generating the RdKW20 WT-*fnr*WT, ∆*dam-fnr*WT, WT-*fnr*GCTC, WT-*fnr*BS*, ∆*dam-fnr*BS*, and WT-*fur*BS* strains.

### Generation of a *H. influenzae* transposon mutant library

A *H. influenzae* RdKW20 transposon mutant library was made as previously described (29), with an estimated number of 30,000 mutants, selected to prevent random loss of mutants with a minimal number of bacteria collected from the lungs at 24 h post-infection (hpi) (~5 × 10^5^ CFU). RdKW20 mutant library stock vial was thawed, grown in 15 mL sBHI with Spec_50_ at 37°C with shaking (225 r.p.m.) to OD_620_ = 0.3, and 1 mL aliquots were stored with 15% glycerol at −80°C for further use. CFU count was determined for a single aliquot by making 10-fold serial dilutions on sBHI agar. Tn-seq data analysis is detailed in the Supplemental Methods.

### Animal procedures

CD1 female mice (18–20 g) aged 4–5 weeks (Charles River Laboratories) were housed under pathogen-free conditions at the IdAB-CSIC animal facility (registration number ES/31-2016-000002-CR-SU-US). When necessary, porcine pancreatic elastase (Elastin Products Company) was intratracheally administered in mice previously anesthetized with isoflurane (Zoetis) for emphysema induction, with matching vehicle solution control groups as described (30). To do so, 10 mg containing 1,350 elastase units (U) were resuspended in 10  mL physiological serum to generate a stock solution (1 mg/mL; i.e., 135 U/mL). To induce emphysema, one 90 µL dose containing 6 elastase U/mouse was administered 21 days before infection.

*H. influenzae* cultures were grown in sBHI under aerobic or, when indicated, anaerobic conditions. For intranasal infection, a 20 µL bacterial suspension was placed at the entrance of the nostrils until complete inhalation by each mouse, previously anesthetized with ketamine (Imalgene, Merial) and xylazine (Rompun, Bayer AG) (3:1). Two assay types were performed:

- *H. influenzae* infection for *in vivo* Tn-seq. Twenty CD1 animals were divided into two groups: mice instilled with phosphate-buffered saline (PBS), *H. influenzae* infected (*n* = 10); and mice with lung emphysema, *H. influenzae* infected (*n* = 10). The previously grown RdKW20 transposon mutant library (see above) was used for infection (20 µL suspension, ~3 × 10^7^ CFU/mouse). Quantification of the inoculated bacterial load was performed through 10-fold dilution in PBS and plating on sBHI agar with Spec_50_. The remaining bacterial inoculum was entirely plated onto five petri dishes (20 cm diameter, 75 mL sBHI agar with Spec_50_ per plate) for collection and processing. Mice in each group were sampled at 24 hpi. Mice were euthanized by cervical dislocation; lungs were aseptically removed, weighed in sterile bags (Stomacher 80, Seward Medical), and homogenized 1:10 (wt/vol) in PBS. For each animal, both lungs were processed together by using 7.5 mL PBS per homogenate. The entire homogenate volume was plated on sBHI agar with Spec_50_ on five petri dishes (20 cm diameter, 1.5 mL homogenate/plate) for further pulling and collection in sBHI with glycerol 15% and freezing. In parallel, 100 µL aliquots of each homogenate were used for serial dilution and plating on sBHI agar with Spec_50_ to determine CFU counts.
- *H. influenzae* infection for competitive index (CI*)* determination*.* Co-infections with WT:mutant, ratio 1:1, were performed. For this purpose, 108 CD1 mice were divided into two groups: mice instilled with vehicle solution (PBS), infected (*n* = 49); and mice with lung emphysema, infected (*n* = 59). Bacteria were grown in sBHI at 37°C in aerobiosis or anaerobiosis to OD_600_ = 0.3 prior to collection. Grown WT and mutant strains were mixed to prepare mixed suspensions (1:1) containing 5 × 10^9^ CFU/mL. Mice were administered 20 µL (~1 × 10^8^ CFU/mouse, 5 × 10^7^ CFU/strain/mouse) by the intranasal route. After 24 h, mice were euthanized by cervical dislocation; lungs were aseptically removed and homogenized as described above. Each homogenate was serially 10-fold diluted in PBS and plated in triplicate on sHTM agar, in the absence or presence of antibiotic (Erm_11_ or Spec_50_, depending on the strain), to determine the number of CFU counts. CI was determined as (CFU_mutant_/CFU_WT_)(output)/(CFU_mutant_/CFU_WT_)(input).

### RNA extraction, purification, and further processing

RNA for sequencing was isolated as follows: *H. influenzae* was grown for 12 h on PVX agar. Two to five colonies were inoculated into 10 mL sBHI, grown for 12 h at 90 r.p.m., diluted into 20 mL fresh sBHI to OD_600_ = 0.05, and grown to OD_600_ = 0.3 at 180 r.p.m. Next, 7 mL bacterial cultures were recovered, pelleted (4,000 r.p.m, 4 min), flash frozen, and stored at −80°C. Bacterial RNA was isolated using the NucleoSpin RNA kit (Macherey-Nagel) (Supplemental Methods). Alternatively, bacterial suspensions collected from anaerobically grown PVX agar plates were normalized to OD_600_ = 0.4 in sBHI; 5 mL aliquots were transferred to 50 mL Falcon tubes with 20 mL sBHI and grown to OD_600_ = 0.3 also in static anaerobiosis. Next, 25 mL bacterial cultures were recovered, pelleted (4,000 r.p.m. for 4 min), flash frozen, and stored at −80°C. Total RNA was isolated using TRIzol reagent (Invitrogen). In both cases, to prevent any DNA interference, a second DNase digestion was performed using RNase-free DNase and cleaning on an RNeasy Mini column (Qiagen). Purified RNA was quantified on a Nanodrop One^C^ (Thermo Fisher Scientific), checked for quality control with RNA 6000 Nano LabChips (Agilent 2100 Bioanalyzer), and sequenced by Admera Health on an Illumina HiSeq platform with 2 × 150 nt reads, estimated 20M PE reads per sample, 10 M in each direction. Three replicates per sample type were sequenced. RNA-seq data analysis is detailed in the Supplemental Methods. RNA-seq raw sequencing data reads were deposited in the National Center for Biotechnology Information Sequence Read Archive and are available under BioProject GSE276728.

RNA for relative quantification by reverse transcription-quantitative PCR (RT-qPCR) was isolated as follows: *H. influenzae* strains were grown in sBHI up to OD_600_ = 0.6 at 180 r.p.m. with 5% CO_2_ or up to OD_600_ = 0.3 in an anaerobic chamber (see above). Next, 7 or 25 mL bacterial cultures were respectively recovered, pelleted (4,000 r.p.m. for 4 min), flash frozen, and stored at −80°C. Total RNA was isolated using TRIzol reagent (Invitrogen) and was quantified on a Nanodrop One^C^. RT-qPCR was performed as indicated in the Supplemental Methods.

### Identification of undermethylated GATC sites in *H. influenzae* genomes

Analysis was performed on raw SMRT sequencing data generated in a previous study (23). The Pacific Biosciences’ SMRT Portal platform (v.2.1.0) was used to identify modQVs at each position. These values were computed as the −10 log (*P* value) based on the distributions of the kinetics of interpulse durations (interpulse duration-IPD ratios). An AmodQV score of 20 is the minimum default threshold and corresponds to a *P* value of 0.01. GATC sites were determined to be undermethylated if below this threshold. A custom Perl script was used to identify undermethylated GATC sites among the GATCs present in the 18 *H*. *influenzae* RdKW20 and COPD clinical isolates genomes. Another Perl script identified pairs or higher-order clusters of GATC motifs separated by <256 nucleotides (31). The Kolmogorov–Smirnov test for two samples, implemented in PAST (32), was used to test whether the distribution of undermethylated GATCs in the *H. influenzae* genomes was homogeneous or heterogeneous.

### Analysis of GATC methylation by quantitative PCR

Genomic DNA was isolated from bacteria using the DNeasy Blood and Tissue kit (Qiagen). DNA samples were separately digested with restriction enzymes with different methylation sensitivities, i.e., *Dpn*I, *Mbo*I, and *SauA*I (New England Biolabs), and purified using DNA, RNA, and protein purification kit (MACHEREY-NAGEL). Digestions were used as templates for quantitative PCR (qPCR), using primer pairs suitable for assessing GATC methylation at each region of interest (Table S3) designed with Primer3 software. To do so, digestion concentrations were normalized to 5.3 ng/µL, and 1 µL/sample was used as template in a 20 µL reaction mixture containing 1× SYBR Premix Ex Taq II (Tli RNaseH Plus). The comparative Ct method was used to obtain relative quantities of DNA that were normalized using the bacteria *gyrA* gene as an endogenous control. The *Y* axes are labeled as enrichment of DNA.

### Identification of DNA sequence motifs for binding of FNR or Fur

The XSTREME tool from the MEME software suite (33) with default settings was used for motif discovery on a set of DNA sequences from the *fnr*, *dms*, *ytfE*, *htpG*, *cydD*, *mts*, *nrf*, *moa*, and *nap* loci, containing 500 bp upstream of each ORF transcription start site. Individual matches to predicted motifs were sought.

### Flow cytometry to monitor expression of transcriptional GFP fusions

Data acquisition was conducted using a Cytomics FC500-MPL (Beckman Coulter, Brea, CA), and subsequent data analysis was performed using FlowJo X (v.10.0.7r) software (Tree Star, Inc., Ashland, OR). Bacteria were grown at 37°C with 5% CO_2_ with shaking (180 r.p.m.), washed, and resuspended in PBS for fluorescence measurement. Fluorescence values for up to 30,000 events were compared with the data from the reporter-less control strain, thus yielding the fraction of ON and OFF cells. For each cell population of interest, the mean fluorescence intensity (MFI) and standard deviation, and the percentage of cells in “ON” and “OFF” states for GFP fluorescence was quantified using FlowJo X (v.10.0.7r) software.

### Fluorescence microscopy

Strains containing GFP fusions were grown on PVX agar at 37°C for 12 h in aerobiosis or anaerobiosis. Next, (i) two to five colonies were inoculated in 10 mL sBHI and incubated at 37°C with 5% CO_2_ for 12 h with shaking (90 r.p.m.); (ii) bacterial suspensions collected from PVX agar anaerobic growth were normalized to OD_600_ = 0.4 in sBHI, and 5 mL aliquots were transferred to tubes with 20 mL sBHI, and grown at 37°C to OD_600_ = 0.3 under static anaerobiosis. In all cases, 250 µL samples were collected by centrifugation at 14,000 r.p.m. for 5 min. Pellets were resuspended in 15 µL of 50% glycerol. One microliter per sample was spotted onto glass coverslips slides, previously coated with 5 µL poly-L-lysine (Merck P4707), and air-dried. Images were captured using a Leica DMi8 fluorescence microscope equipped with a Leica HCX PL APO ×100/1.40–0.70 oil objective, a Hamamatsu ORCA Flash 4.0 LT camera, and the LAS X software. Images were analyzed using the Icy software (v.2.4.2.0); fluorescence intensity was quantified in 1,000 bacteria per sample type, in at least 10 independent images per sample.

### Statistical analyses

Statistical analyses are detailed in each figure legend. In all cases, a *P* value of <0.05 was considered statistically significant. Analyses were performed using Prism software (v.7 for Mac, GraphPad Software) statistical package. If not otherwise indicated, all experiments were performed in three replicates.

## RESULTS

### Genome-wide transposon mutagenesis identifies Dam methyltransferase contribution to *H. influenzae* pulmonary infection

**Fig 1:**
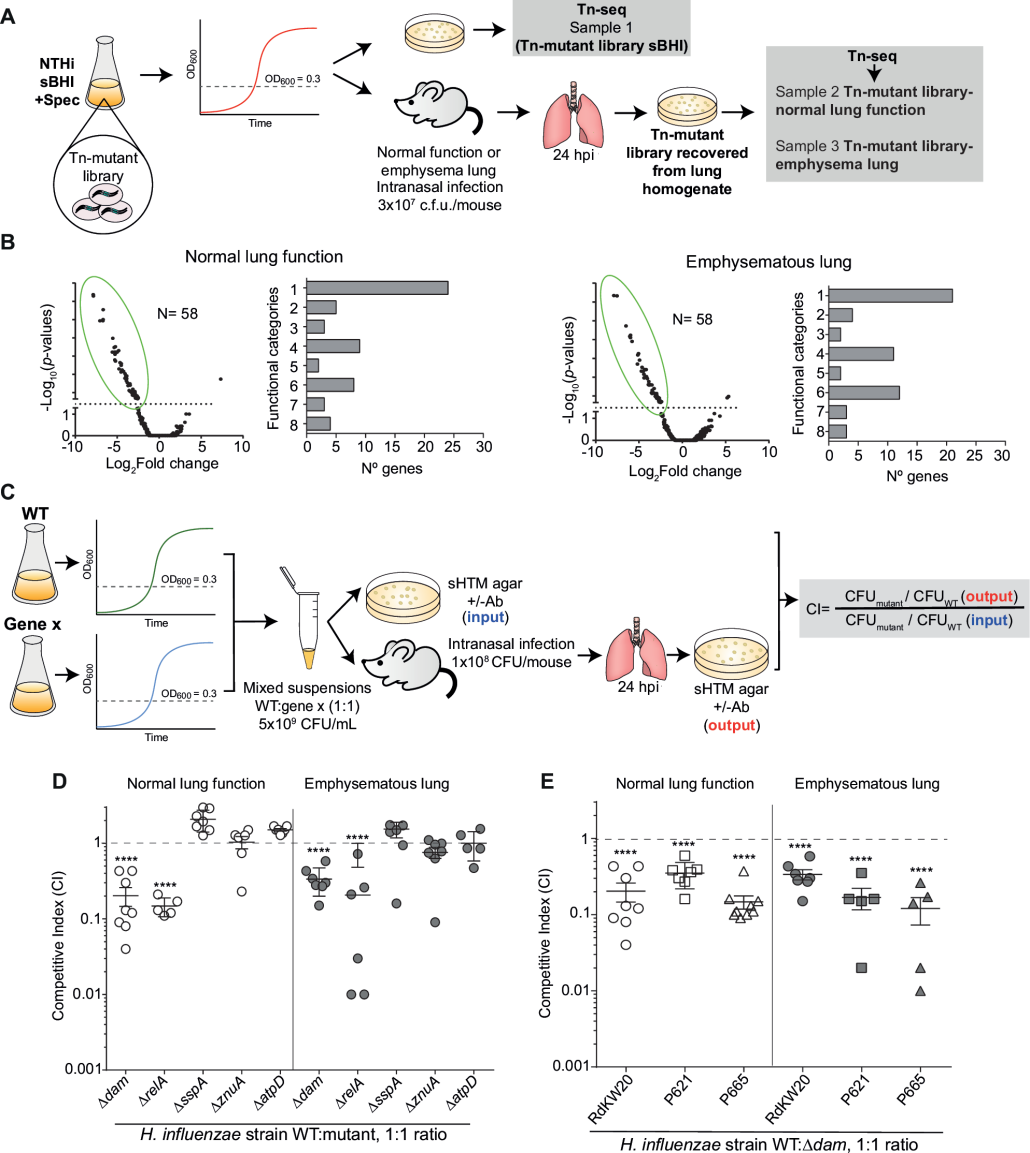
*In vivo* Tn-seq reveals *H. influenzae* genes required for survival in murine lungs. (**A**) Schematic representation of *H. influenzae* sample generation for *in vivo* Tn-seq analysis. (**B**) Volcano plot showing fold change of bacterial under- and overrepresented genes when comparing “output versus input” libraries. Upon infection of mice with normal lung function, 58 *H. influenzae* genes were underrepresented, compared to *in vitro* growth in sBHI (left panel). In mice with lung emphysema, another 58 *H. influenzae* genes were underrepresented compared to sBHI growth (right panel). Data were manually analyzed to define systems underrepresented *in vivo*. Organization of underrepresented genes into functional categories: 1, metabolism; 2, transport systems; 3, nucleic acid processing; 4, transcription and translation; 5, stress response/nutrient starvation; 6, cell wall; 7, others; and 8, hypothetical proteins. (**C**) Schematic representation of *H. influenzae* mice airway infection for competitive index (CI) determination. (**D**) RdKW20 WT and mutant strains were exponentially grown in sBHI. Mice were intranasally infected with bacterial mixed suspensions (WT:mutant, ratio 1:1). Mice were euthanized at 24 hpi; lungs were processed, serially diluted 10-fold in PBS, and plated on sHTM agar, in the absence and presence of antibiotic. CFU counts were used for CI determination. (**E**) *H. influenzae* RdKW20, P621, and P665 WT and ∆*dam* strains were exponentially grown in sBHI. CD1 mice were intranasally infected with bacterial mixed suspensions; mice were euthanized at 24 hpi, and lungs were processed for CI determination. In panels D and E, statistically significant differences were determined by *t*-test. ****, *P* < 0.0001.

Tn-seq was first employed to measure the *in vivo* fitness consequences of insertional mutagenesis by transposons into the *H. influenzae* RdKW20 strain using a mouse model of lung infection (30, 34) (Fig. 1A). By making use of its high natural transformation frequency, we created a saturated transposon library in the RdKW20 strain. A library of 30,000 transposon mutants was exponentially grown in sBHI (sample 1, referred to as input mutant library), and used for murine lung infection. Animals with normal lung function or with lung emphysema were employed. Lung homogenates were recovered from infected mice at 24 hpi to generate samples 2 and 3 (output mutant libraries). Samples 1, 2, and 3 were processed for Tn-seq, and mutant abundance was profiled. Essential web-based interface for Tn-seq data analysis (35) was used to identify essential and conditionally essential genes required for survival in mice, in comparison to sBHI medium. A total of 368 million reads were retrieved for the sequence run with the 24 hpi and the input library, which contained between 9 and 45 million reads per sample. Transposon mutants were identified in 1,259–1,271 (dependent on the group comparisons) of the 1,765 annotated genes; no transposon mutants were identified in ~500 genes.

Transposon insertions were significantly underrepresented in 58 genes after infection of mice (normal lung function) with the Tn-seq library, indicating that loss of these genes’ functions reduces *in vivo* fitness during lung infection. Many of these genes are involved in bacterial metabolism, stress responses, transport, transcription and translation, and cell wall integrity. Lung emphysema is a frequent pathophysiological trait in COPD patients (1). Infection of mice undergoing lung emphysema (30) with the same transposon mutant library also identified 58 genes with underrepresented transposon tags, with a core set of 38 genes required for lung infection in both settings (Fig. 1B; Data Set S1, Fig. S1A). These results suggest that bacterial countermeasures against host defenses remain critical for infection independently of lung function status.

Several genes such as *galU* and *galE* have previously been demonstrated to contribute to *H. influenzae* lung infection (36, 37). Furthermore, 33 genes were listed in a previous *in vivo* genome-wide transposon-based (high-throughput insertion tracking by deep sequencing [HITS]) screen (36). Interestingly, the *dam* gene was underrepresented in both our Tn-seq experiment and the previous HITS screening (Fig. S1), supporting that Dam methyltransferase activity may contribute to *H. influenzae* survival within the airways.

In direct comparisons of the WT RdKW20 strain and isogenic Δ*dam*, Δ*relA*, Δ*sspA*, Δ*znuA*, and Δ*atpD* mutant derivatives, the RdKW20Δ*dam* and RdKW20Δ*relA* strains showed significant reductions in competitive fitness during *in vivo* murine lung infection (Fig. 1C and D). Attenuation was not observed for the *sspA*, *znuA* and *atpD* mutant strains.

Lower competitive fitness was also observed when comparing WT and Δ*dam* mutants of two COPD respiratory isolates, strains P621 and P665 (23) (Fig. 1E; Fig. S2A). These results expand on previous studies supporting a role for Dam methyltransferase in invasive infection (22), and also show its importance to pulmonary infection. The role of Dam methyltransferase epigenetic control on *H. influenzae* lung infection was next examined.

### DNA methylome analysis identifies GATC undermethylation within putative regulatory regions

**Fig 2:**
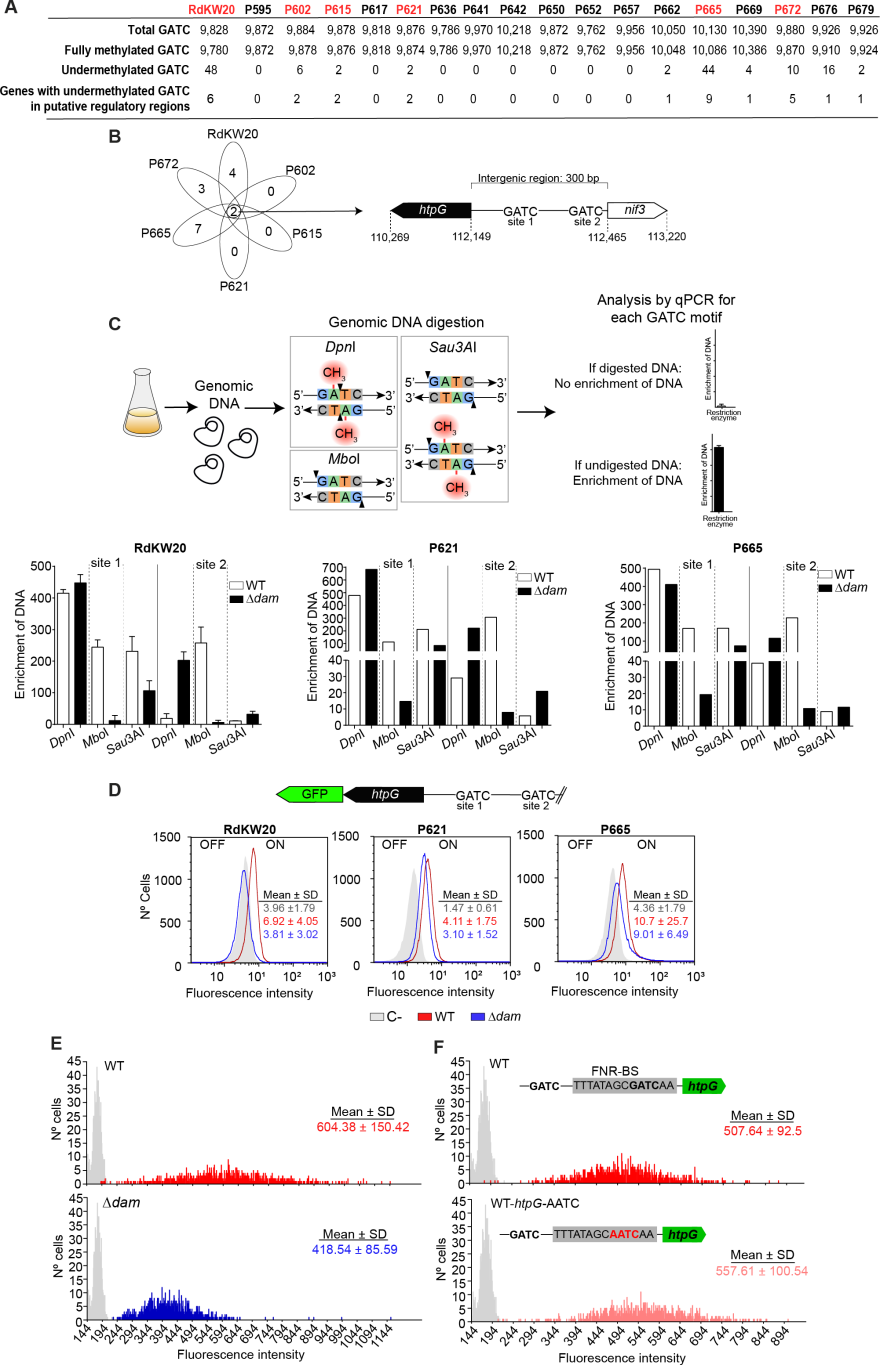
*H. influenzae* DNA methylome analysis by using SMRT sequencing data. (**A**) Total, fully methylated, and undermethylated number of GATC sites genome-wide, and genes with undermethylated GATC motifs in their putative regulatory regions across 18 *H. influenzae* genomes, corresponding to the RdKW20 reference strain and 17 COPD respiratory isolates. (**B**) Venn diagram summarizing strain-specific and commonly found genes with undermethylated GATC sites in putative regulatory regions in the RdKW20, P602, P615, P621, P665, and P672 genomes. Schematic representation of the *htpG-nif3* intergenic region where two GATC sites are located, named sites 1 and 2. (**C**) Analysis of GATC motif methylation on sBHI aerobically grown bacterial cultures. Purified gDNA samples were digested with restriction enzymes with different methylation sensitivities: *Dpn*I, methylated restriction site; *Mbo*I, unmethylated restriction site; *SauA*I, methylated and unmethylated restriction site. Digested DNA was used as a template for qPCR performed using specific primer pairs for each GATC motif. Methylation pattern of GATC sites 1 and 2 in the intergenic region of *htpG-nif3* in RdKW20 (left), P621 (middle), and P665 (right) WT and ∆*dam* strains carrying a chromosomal *htpG::gfp* transcriptional fusion. RdKW20 strain, used as negative control for fluorescence. (**D**) Flow cytometry analysis of *htpG* gene expression during mid-exponential bacterial aerobic growth. RdKW20 (left), P621 (middle), and P665 (right) WT and ∆*dam* strains carrying a chromosomal *htpG::gfp* transcriptional fusion were used. RdKW20 strain, used as negative control for fluorescence. (**E**) Fluorescence microscopy analysis of *htpG* gene expression during mid-exponential bacterial aerobic growth of the RdKW20 WT and ∆*dam gfp* reporter strains. Images captured using a fluorescence microscope were analyzed by quantifying fluorescence in a total of 1,000 cells/sample type. Statistically significant differences were determined by one-way analysis of variance (ANOVA) (Kruskal-Wallis test) (WT vs ∆*dam*). ****, *P* < 0.0001. (**F**) Fluorescence microscopy analysis of *htpG* gene expression during mid-exponential bacterial aerobic growth of the RdKW20 WT and WT-*htpG*-AATC *gfp* reporter strains. Fluorescence quantification was carried out as in panel** E**. Statistically significant differences were determined by one-way ANOVA (Kruskal-Wallis test); differences were not observed.

We first conducted PacBio SMRT sequencing methylome analysis to identify the Dam methylation status at all GATC motifs in the RdKW20 genome. Second, we used PacBio SMRT data generated in a previous study (23) to infer the methylation state of GATC sites in the chromosome of 17 non-typeable *H. influenzae* isolates collected from COPD sputum samples. Genomes from clinical isolates contained variable numbers of GATC motifs, from 9,762 to 10,390, most of them fully methylated on both strands (Fig. 2A). Next, we screened for undermethylated DNA targets within predicted regulatory elements to identify transcriptional units that may be influenced by Dam methylation status. In the RdKW20 genome, of 9,828 GATC motifs, 48 were undermethylated; from those, 6 are located in putative regulatory regions (Fig. 2A). By contrast, 8 of 17 clinical COPD isolates contained no detectable undermethylated sites; the remainder had from one to nine genes with detectable undermethylated GATC sites within putative regulatory regions.

Only a single undermethylated GATC site was shared by multiple strains, which was found within a 300 bp intergenic region located between the divergently transcribed *htpG* and *nif3* genes (Fig. 2B). This site was seen in RdKW20 and in five of the COPD isolates (P602, P615, P621, P665, and P672) (Data Set S2, sheet 1). The *htpG* gene is predicted to encode the molecular chaperone high-temperature protein G (HtpG), a bacterial homolog of eukaryotic heat shock protein 90 (Hsp90) (38). The *nif3* gene is predicted to encode an Ngg1p interacting factor 3-like protein with unknown function. Two GATC sites are present between the *htpG* and *nif3* genes. The *htpG-*proximal site 1 shows undermethylation by SMRT sequencing, whereas site 2 closer to *nif3* remains fully methylated (Fig. 2B). To confirm the methylome-based difference, methyl-sensitive and insensitive restriction enzymes were used in combination with qPCR. Results by this assay supported Dam methylation at GATC site 2 but not at GATC site 1 for the RdKW20, P621, and P665 strains (Fig. 2C).

Gene expression of *htpG* and *nif3* was next investigated in aerobically grown bacterial cultures by flow cytometry using transcriptional fusions with the *gfp* gene (Fig. S2C). Gene expression was similar for *nif3* in WT and ∆*dam* strains of RdKW20, so this locus was excluded from further analysis (Fig. S3). The *htpG::gfp* reporter showed heterogeneous expression with an MFI of 6.92, and 20.7% of cells were classified as “ON state.” In contrast, fluorescence of the ∆*dam* strain was lower with an MFI of 3.81 and only 1.67% of ON state cells, close to the non-GFP WT control strain (MFI = 3.96). These data suggest that GATC methylation status affects transcriptional activity. Strain RdWK20 observations were supported by experiments made on COPD strains P665 and P621, with decreasing MFI and percentage of ON state cells for the respective ∆*dam* mutants (Fig. 2D). Flow cytometry results were further supported by fluorescence microscopy and quantification of fluorescence at the single bacterial cell level. Expression of the *gfp* fusion in the *htpG* locus was heterogeneous with different proportions of cells containing variable levels of fluorescence intensity. The distribution of single-cell GFP fluorescence intensities among cells showed a significant decrease in the Δ*dam* strain (418.54 ± 85.59) compared to WT (604.38 ± 150.42) (Fig. 2E).

As described above, methylation of the GATC site 1 was not demonstrated by the restriction-qPCR assay (Fig. 2C). To delve deeper into the relationship between the GATC site 1 and the *htpG* expression phenotype, a chromosomal modification of this GATC motif was constructed. Results showed that the fluorescence intensity of the WT-*htpG*-AATC strain (557.61 ± 100.54) was comparable to that of the isogenic WT strain (507.64 ± 92.50) (Fig. 2F). Therefore, epigenetic regulation of *htpG* expression seems to involve more than just methylation at GATC site 1. Undermethylation at this site strongly suggests a methylation-blocking effect, potentially mediated by the binding of an unknown protein in such a way that the interplay between Dam methylation and site-specific blocking of GATC motifs may collectively contribute to the observed phenotypic outcomes.

### Transcriptome profiling shows downregulation of the FNR regulon by Dam methylation

**Fig 3:**
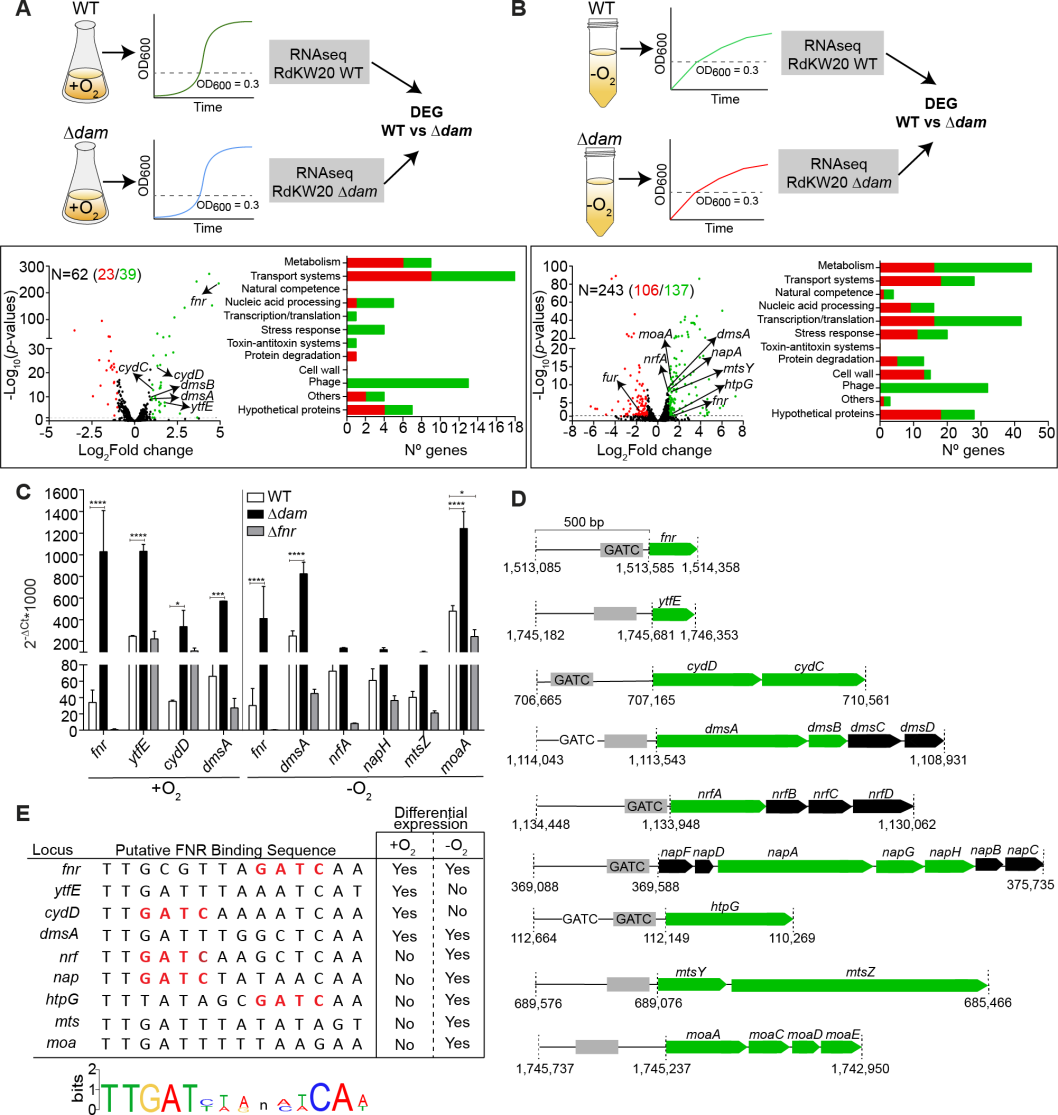
Upregulation of the FNR regulon gene expression upon *dam* inactivation in *H. influenzae* aerobic and anaerobic cultures. (**A**) RdKW20 WT and ∆*dam* strains were exponentially grown in sBHI in aerobiosis, and samples were processed for RNA-seq and differential gene expression analysis. Volcano plot representing the fold change of differentially expressed genes (DEGs) upon *dam* inactivation, leading to upregulation of 39 and downregulation of 23 genes (*P* < 0.05). A proportion of the DEGs encode products involved in bacterial metabolism and transport of iron and heme (upregulated in RdKW20∆*dam*: *hgpB*, *hitA*, *hgpD*, *hgpC*, *hfeB*, and *hfeA*; downregulated in RdKW20∆*dam*: *hxuB*, *ftnB*, and *ftnA*). Organization of DEGs into functional categories (right panel): green, upregulated upon Dam inactivation; red, downregulated upon Dam inactivation. (**B**) WT and ∆*dam* strains of RdKW20 were exponentially grown in sBHI in anaerobiosis, and samples were processed for RNA-seq and differential gene expression analysis. Volcano plot representing the fold change of DEG in ∆*dam* under anaerobic growth, leading to upregulation of 137 and downregulation of 106 genes (*P* < 0.05). Organization of DEG genes into functional categories stated as in panel** A**. (**C**) Expression of the *fnr*, *ytfE*, *cydD*, *dmsA*, *nrfA*, *napH*, *mtsZ*, and *moaA* genes, under aerobic (left) or anaerobic (right) conditions, determined by RT-qPCR. Significant differences determined by analysis of variance with Tukey’s multiple comparison test. *, *P* < 0.05; ***, *P* < 0.001; ****, *P* < 0.0001. (**D**) Genomic organization of the *fnr*, *ytfE*, *cyd*, *dms*, *nrf*, *nap*, *htpG*, *mts*, and *moa* loci showing the relative position of GATC and predicted FNR binding sites (gray box) in their putative regulatory regions. DEGs, upregulated upon Dam inactivation, are shown in green. (**E**) List of genes that belong to the FNR regulon and are differentially expressed (upregulated) in the ∆*dam* strain. A sequence logo shows residues of the *H. influenzae* predicted FNR binding site (FNR-BS) consensus sequence, and the FNR-BS predicted for the *fnr*, *ytfE*, *cydD*, *dmsA*, *nrf*, *nap*, *htpG*, *mts*, and *moa* loci. In the right columns, growth conditions where differential gene expression was found by RNA-seq are shown.

To further determine the consequences of Dam methylation on global gene expression, we sequenced the transcriptomes of exponentially grown *H. influenzae* WT and Δ*dam* strains in the RdKW20 background (Fig. 3A, aerobic growth). The biggest change found in the Δ*dam* mutant was upregulation of the *fnr* gene, which encodes the FNR transcriptional regulator. FNR is an oxygen-sensitive master regulator of the switch to anaerobic growth, known to be involved in *H. influenzae* defense against reactive NO donors (12, 38, 39). FNR directly senses oxygen by virtue of its iron-sulfur center, which, under oxygen limitation, promotes its dimerization, DNA binding, and transcriptional activation of target genes. Upon oxidation, the iron-sulfur center undergoes a transition that converts FNR to its inactive monomeric form (40). Expression of the *fnr* gene was upregulated in the Δ*dam* strain, as were the *ytfE*, *cydDC*, and *dmsAB* genes, which are known parts of the FNR regulon (Data Set S2, sheet 2, highlighted in green; Fig. 3C, +O_2_). The *ytfE* gene encodes a putative di-iron protein that repairs nitrosative damage; the *dmsABCD* genes encode a sulfoxide reductase essential for *H. influenzae* infection; CydDC transporters export glutathione and cysteine to the periplasm, important for tolerance to NO (12, 38, 41).

These findings were unexpected as RNA-seq of aerobic cultures identified a connection between Dam methylation and the FNR regulon. Such aerobic growth likely uncoupled epigenetic regulation and FNR activity, maybe underestimating its consequences. We next sequenced the transcriptomes of WT and Δ*dam* strains during growth in anaerobic conditions (Fig. 3B; Data Set S2, sheet 3; Fig. S4). Upregulation of genes belonging to the FNR regulon was again observed, including *fnr* and *dmsA*, and also the *nrfA* nitrite reductase and the *napAGHB* nitrate reductase encoding genes (12, 38). Previous reports included the *htpG* (see above) and *mtsZ* (encoding a methionine sulfoxide reductase) (42) genes as members of the FNR regulon (38), and these genes were also upregulated in the Δ*dam* strain upon anaerobic growth. Moreover, DmsA, NapA, and MtsZ are molybdopterin-containing enzymes, and the molybdopterin biosynthetic genes (*moaEDC* and *mog*) were also upregulated in the Δ*dam* strain (Data Set S2, sheet 3, genes belonging to the FNR regulon highlighted in green). We further confirmed that these genes had increased expression in the Δ*dam* strain and are members of the FNR regulon, since their expression was decreased in Δ*fnr* compared to the WT strain (Fig. 3C, −O_2_).

These results showed that inactivation of the *dam* gene leads to increased expression of genes belonging to the FNR regulon, including the *fnr* gene itself, indicating that Dam methylation activity represses expression of the FNR regulon.

### Dam methyltransferase epigenetic control of the FNR regulon gene expression: the *dmsA* and *htpG* cases

By checking the presence of GATC motifs within 500 bp upstream of each differentially expressed gene, we identified such motifs in the intergenic regions upstream of multiple genes differentially expressed in the Δ*dam* strain compared to WT, including genes belonging to the FNR regulon, i.e., within the *fnr*, *cydDC*, *dmsABCD*, *nrfABCD*, *napFDAGHBC*, and *htpG* intergenic regions (Fig. 3D; Data Set S2, sheets 2 and 3, column I). Using the previously established FNR binding consensus sequence (12), we predicted FNR binding sites (FNR-BS) in the *fnr*, *ytfE*, *cydDC*, *dmsABCD*, *nrfABCD*, *napFDAGHBC*, *htpG*, *mtsYZ*, and *moaACDE* promoter-regulatory regions. Moreover, we found an overlap between the GATC motifs and predicted FNR-BS present within the promoter-regulatory regions of the *fnr*, *cydDC*, *nrfABCD*, *napFDAGHBC*, and *htpG* genes (Fig. 3D and E).

**Fig 4:**
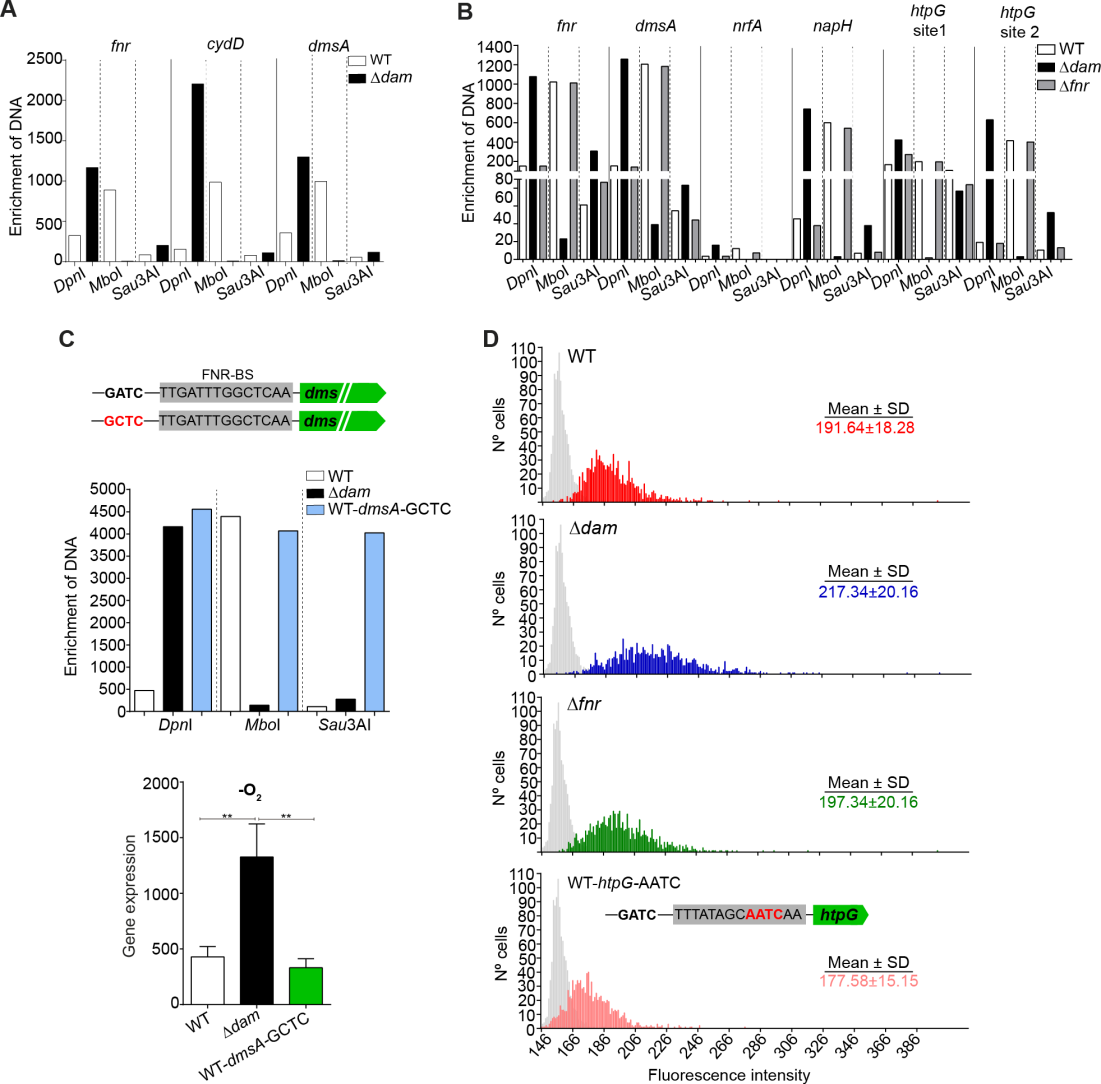
Regulatory effects of Dam methylation on FNR regulon gene expression. (**A**) Methylation in GATC motifs located in the promoter regions of the *fnr*, *cydD*, and *dmsA* genes. Aerobically grown cultures of RdKW20WT and ∆*dam* strains were used, which showed methylation of all three GATC motifs. (**B**) Methylation in GATC motifs located in the putative promoter region of the *fnr*, *dmsA*, *nrfA*, *napH*, and *htpG* genes. Anaerobically grown cultures of RdKW20WT, ∆*dam*, and ∆*fnr* strains were used, which showed methylation of all tested motifs, except the *htpG* GATC site 1. (**C**) Analysis of GATC methylation in the promoter region of the *dmsA* gene. Anaerobically grown RdKW20 WT-*dmsA-*WT, Δ*dam-dmsA-*WT, and WT-*dmsA-*GCTC strains were used (upper panel). RNA was isolated from anaerobically grown cultures of the RdKW20 WT-*dmsA-*WT, Δ*dam-dmsA-*WT, and WT-*dmsA-*GCTC strains. Expression of the *dmsA* gene was determined by RT-qPCR (lower panel). (**D**) Fluorescence microscopy analysis of *htpG* expression during anaerobic growth of the RdKW20 WT, ∆*dam*, ∆*fnr* and WT-*htpG*-AATC *gfp* reporter strains. Images captured using a fluorescence microscope were analyzed by quantifying fluorescence in a total of 1,000 cells per sample type. Statistically significant differences were determined by one-way ANOVA (Kruskal-Wallis test). ****, *P* < 0.0001.

Methylation was confirmed by restriction-qPCR assay for all these GATC motifs, except for the *htpG* GATC site 1 (Fig. 2C; Fig. 4A and B). Moreover, GATC methylation data were comparable for WT and Δ*fnr* strains when grown in anaerobic cultures, suggesting that FNR is unlikely to interfere with Dam activity at the *fnr*, *dms*, *nrf*, *nap*, and *htpG* (site 2) upstream regions (Fig. 4B). Methylation of the *htpG* GATC site 1 could not be confirmed in the Δ*fnr* mutant (Fig. 4B), suggesting the existence of additional unknown elements likely blocking such methylation.

Our data suggest that epigenetic control indirectly affects the expression of *ytfE*, *mts*, and *moaACDE*, since these genes lack GATC sites in their upstream regions. For the rest of the genes identified, methylation effects may be direct or indirect, since they all contain upstream GATC sites but could also be coupled to additional regulatory motifs due to the presence of potential FNR-BS (*fnr*, *cydDC*, *dmsABCD*, *napAGHB*, and *nrfA*), which sometimes overlap with the GATC motif (*fnr*, *cydDC*, *napAGHB*, and *nrfA*) (Fig. 3D).

To directly assess if methylation of a GATC motif non-overlapping with a predicted FNR-BS affects gene expression, we engineered a GATC to GCTC mutation in the motif located in the *dmsA* gene regulatory region. The absence of growth defects and lack of methylation in the RdKW20 WT-*dmsA-*WT, Δ*dam-dmsA-*WT, and WT-*dmsA-*GCTC strains were confirmed (Fig. 4C, upper panel; Fig. S2D). Anaerobic *dmsA* gene expression in the WT-*dmsA-*GCTC strain was comparable to that in the isogenic WT and lower than that in the Δ*dam* strain (Fig. 4C, lower panel), excluding a direct role for such GATC motif in the observed epigenetic control.

Conversely, to assess if methylation of a GATC motif overlapping with a predicted FNR-BS affects gene expression, we next investigated *htpG* gene expression in anaerobic cultures by using the *htpG::gfp* fusion strain described above. Expression of *htpG* was heterogeneous, and in contrast to observations made in aerobiosis, the Δ*dam* strain (217.34 ± 20.16) had a significantly higher mean fluorescence intensity than WT (191.64 ± 18.28) (***, *P* < 0.0001) (Fig. 4D). This observation is in agreement with our anaerobic RNA-seq data (Fig. 3; Data Set S2, sheet 3). We also assessed if FNR has a regulatory role on *htpG* expression, but mean fluorescence intensity by the Δ*fnr* strain (197.34 ± 20.16) was comparable to that of the WT (Fig. 4D). Unexpectedly, chromosomal modification of the *htpG* GATC site 1 showed a significant decrease in GFP expression, compared to WT (mean fluorescence intensity, 177.58 ± 15.15 compared to 191.64 ± 18.28, (***, *P* < 0.0001) (Fig. 4D, bottom panel). These results highlight the complexity of *htpG* regulation, where Dam methylation, site-specific blocking of GATC motifs, and FNR may collectively contribute to the observed phenotypes under anaerobic growth: the observed GATC site 1 undermethylation suggests a methylation-blocking effect by the binding of unknown proteins, or maybe by the sequences flanking this GATC site (43), which could also hinder possible FNR effects; overexpression of *fnr* upon Dam inactivation may overcome such hindrance and support FNR contribution to the observed regulation of *htpG* expression.

These results support a key role for epigenetic regulation of the FNR regulon, also highlighting the need for seeking regulation of FNR itself to reach further understanding.

### Dam methylation and Fur control the expression of FNR and its regulon

**Fig 5:**
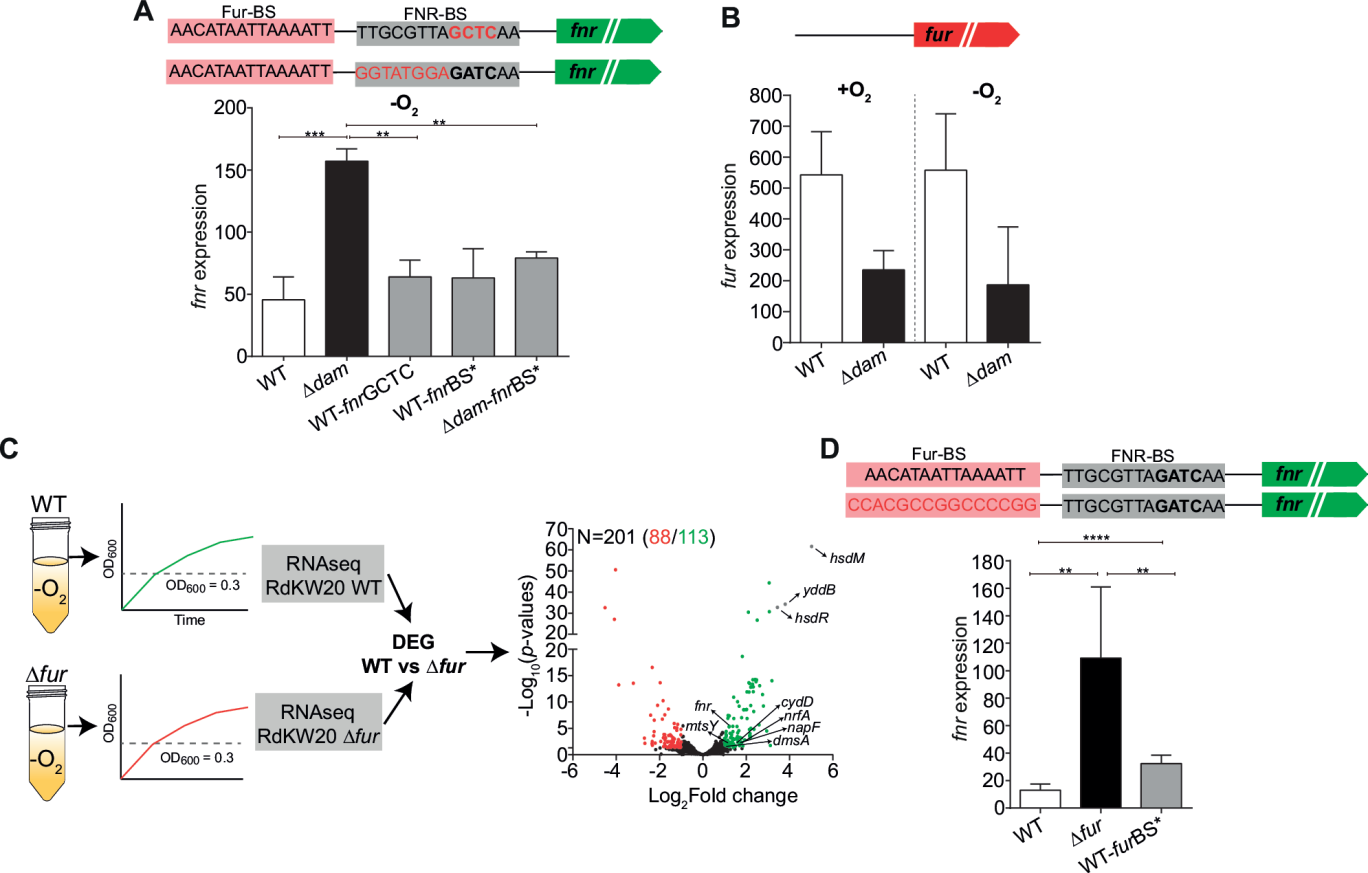
Dam methylation and Fur control the expression of FNR and its regulon. (**A**) Expression of the *fnr* gene, measured by RT-qPCR. RdKW20 WT-*fnr*WT, ∆*dam-fnr*WT, WT-*fnr*GCTC, WT-*fnr*BS*, and ∆*dam-fnr*BS* were grown in anaerobiosis; total RNA was isolated; and *fnr* expression was measured. Chromosomal modifications are indicated in red text. (**B**) Total RNA was isolated from aerobic and anaerobically grown RdKW20 WT and ∆*dam* bacterial cultures, and *fur* expression was quantified by RT-qPCR. (**C**) RdKW20 WT and ∆*fur* strains were exponentially grown in sBHI in anaerobiosis, and samples were processed for RNA-seq and differential gene expression analysis. Volcano plot representing the fold change of DEG in the ∆*fur* strain, leading to upregulation of 113 genes and downregulation of 88 genes (*P* < 0.05). Most upregulated genes (*hsdM*, *yddB*, and *hsdR*) are labeled in gray; upregulated genes belonging to the FNR regulon (*fnr*, *mtsY*, *cydD*, *nrfA*, *napA*, and *dmsA*) are also highlighted. (**D**) Expression of the *fnr* gene in the WT, Δ*fur,* and WT-*fur*BS* strains, grown in the absence of oxygen, by RT-qPCR. In panels **A** and **D**, regulatory regions with GATC motifs (bold) and predicted FNR (gray boxes) or Fur (red boxes) binding sites are shown. Statistically significant differences were determined by analysis of variance with Tukey’s multiple comparison test (**A**) or *t*-test (**B and D**). **, *P* < 0.01; ***, *P* < 0.001; ****, *P* < 0.0001.

To directly test how Dam methylation and FNR binding affect the expression of *fnr* itself, we next engineered a GATC to GCTC mutation within the predicted FNR-BS located in the *fnr* regulatory region. The absence of growth defects and lack of methylation in the WT-*fnr-*GCTC strain were confirmed (Fig. S2E and Fig. S5). Anaerobic *fnr* gene expression in WT-*fnr-*GCTC was comparable to that in the WT strain, and lower than that in the Δ*dam* strain (Fig. 5A). Chromosomal modification of the putative FNR binding site (5′-TTGCGTTAGATCAA to 5′-GGTATGGAGATCAA, strain WT*-fnr*BS*) preserved GATC methylation (Fig. S5) but did not modify *fnr* gene expression. However, introducing this change in the Δ*dam* strain to generate the Δ*dam-fnr*BS* strain lowered *fnr* gene expression when compared to that observed in the Δ*dam* strain (Fig. 5A).

These results suggest that epigenetic control of *fnr* expression is not directly associated with methylation of the GATC site within the *fnr* intergenic region but may instead involve additional regulatory elements. We hypothesized that such elements may, in turn, be positively regulated by methylation and have a repressor effect on *fnr* gene expression. If so, *dam* knockout would reduce repressor expression and contribute to increased *fnr* expression, as observed when comparing WT and Δ*dam* strains. Also, decreased *fnr* gene expression when comparing Δ*dam* and Δ*dam-fnr*BS* suggests that in the absence of repressor, FNR would positively regulate its own expression through the predicted FNR binding site.

We next used the XSTREME tool from the MEME software suite to scan the *fnr* promoter region. An individual match for a Fur binding motif was predicted (sequence: 5′-AACATAATTAAAATT). Our anaerobic RNA-seq data showed downregulation of the *fur* gene expression in the Δ*dam* strain (Data Set S2, sheet three highlighted in red; Fig. 5B). A regulatory interplay between Fur and FNR has been previously suggested in *E. coli*, with a repressor effect of Fur on FNR (44). We analyzed the Fur regulon in anaerobically grown RdKW20 cultures by comparing differential gene expression of the WT and Δ*fur* strains. Notably, *fnr* and several genes belonging to its regulon (*cydD*, *nrf* locus, *napF* locus, *dms* locus, and *mtsZ*) were shown to be upregulated in the Δ*fur* strain (Fig. 5C; Data Set S2, sheet 4, highlighted in green). Overexpression of *fnr* in the Δ*fur* strain was further confirmed; moreover, chromosomal modification of the putative Fur binding site (5′-AACATAATTAAAATT to 5′-CCACGCCGGCCCCGG, strain WT*-fur*BS*) also increased expression of the *fnr* gene compared to that in the isogenic WT strain (Fig. 5D). Detailed analysis of the putative *fur* promoter-regulatory region did not show GATC motifs, limiting a direct analysis of *fur* expression and Dam epigenetic control.

Thus, the expression of *fnr* in *H. influenzae* is negatively regulated by Fur, whose expression, in turn, is upregulated by Dam methylation. This regulatory network operates independently of oxygen availability in terms of gene expression, although it should be functional under low-oxygen conditions to allow for FNR active conformation, thus likely uncoupling gene expression from protein activity.

### Dam methyltransferase and the FNR regulator contribute to *H. influenzae* survival within the host lungs

**Fig 6:**
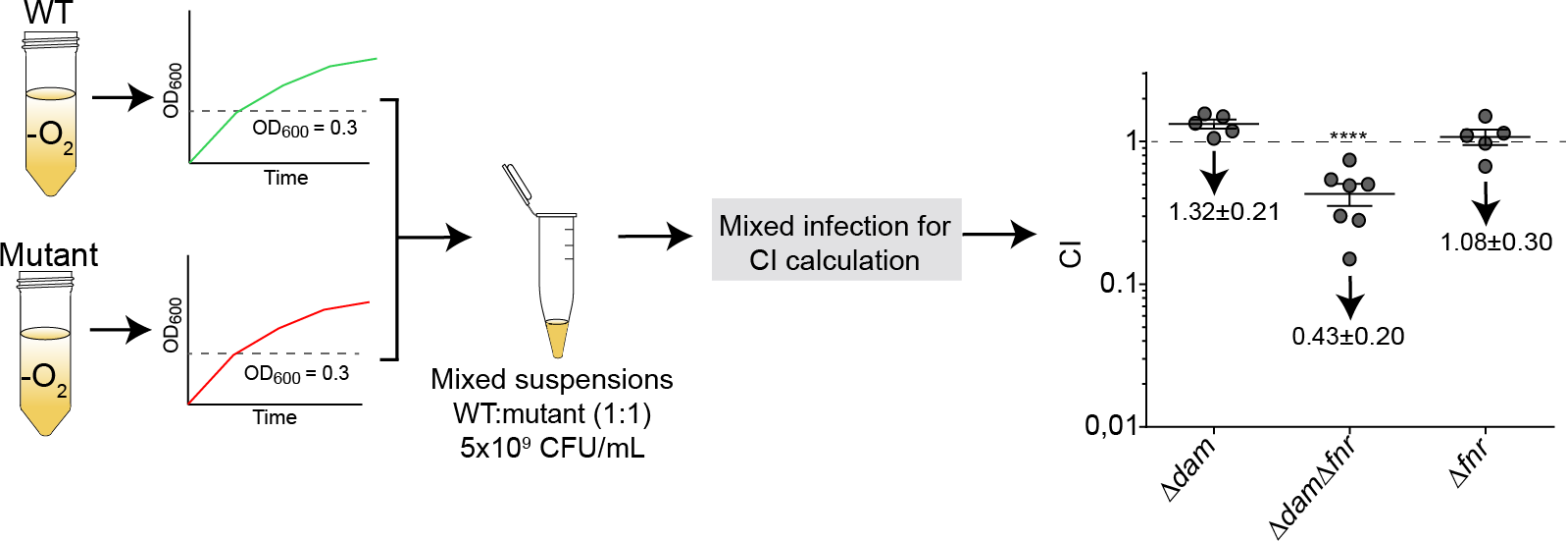
Growth conditions of the *H. influenzae* strains used to prepare the mice infecting inocula have an effect on bacterial *in vivo* lung survival. RdKW20 WT and mutant strains were grown in anaerobiosis. Mice were intranasally infected with mixed suspensions, WT:mutant, ratio 1:1. At 24 hpi, mice were euthanized, lungs were processed, and lung homogenates were serially diluted and plated on sHTM agar, in the absence and presence of antibiotic. CFU counts were used for CI determination. Statistically significant differences were determined by *t*-test. ****, *P* < 0.0001.

The starting point of this study is the finding that Dam contributes to *H. influenzae* airway infection. In the assays shown in Fig. 1, murine infection was performed with aerobically grown bacteria. In these conditions, attenuation by the Δ*dam* mutant strain may involve *fnr* overexpression but FNR non-active conformation, and bacterial aerobic cultures are likely to behave as a Δ*dam*Δ*fnr* double mutant in practical terms. Thus, we speculated that infection of mice with anaerobically grown Δ*dam* bacteria may not necessarily lead to attenuation since FNR would be active and its regulon expressed, contributing to bacterial evasion of stress within diseased alveoli with hypoxia. Indeed, this was the case when mice with a previously developed emphysema lesion (i.e., damaged lung tissue) were infected with anaerobically grown WT and Δ*dam* strains mixed together (Fig. 6). Following this rationale, WT mixed infection with a Δ*dam*Δ*fnr* double mutant strain had clear attenuation. However, this does not mean that FNR is the only contributor to *in vivo* survival of anaerobically grown infecting bacteria since Δ*fnr* was not attenuated on its own (Fig. 6). In sum, Dam-mediated epigenetic control and the FNR regulator synergistically contribute to *H. influenzae* survival within the murine lungs.

## DISCUSSION

**Fig 7:**
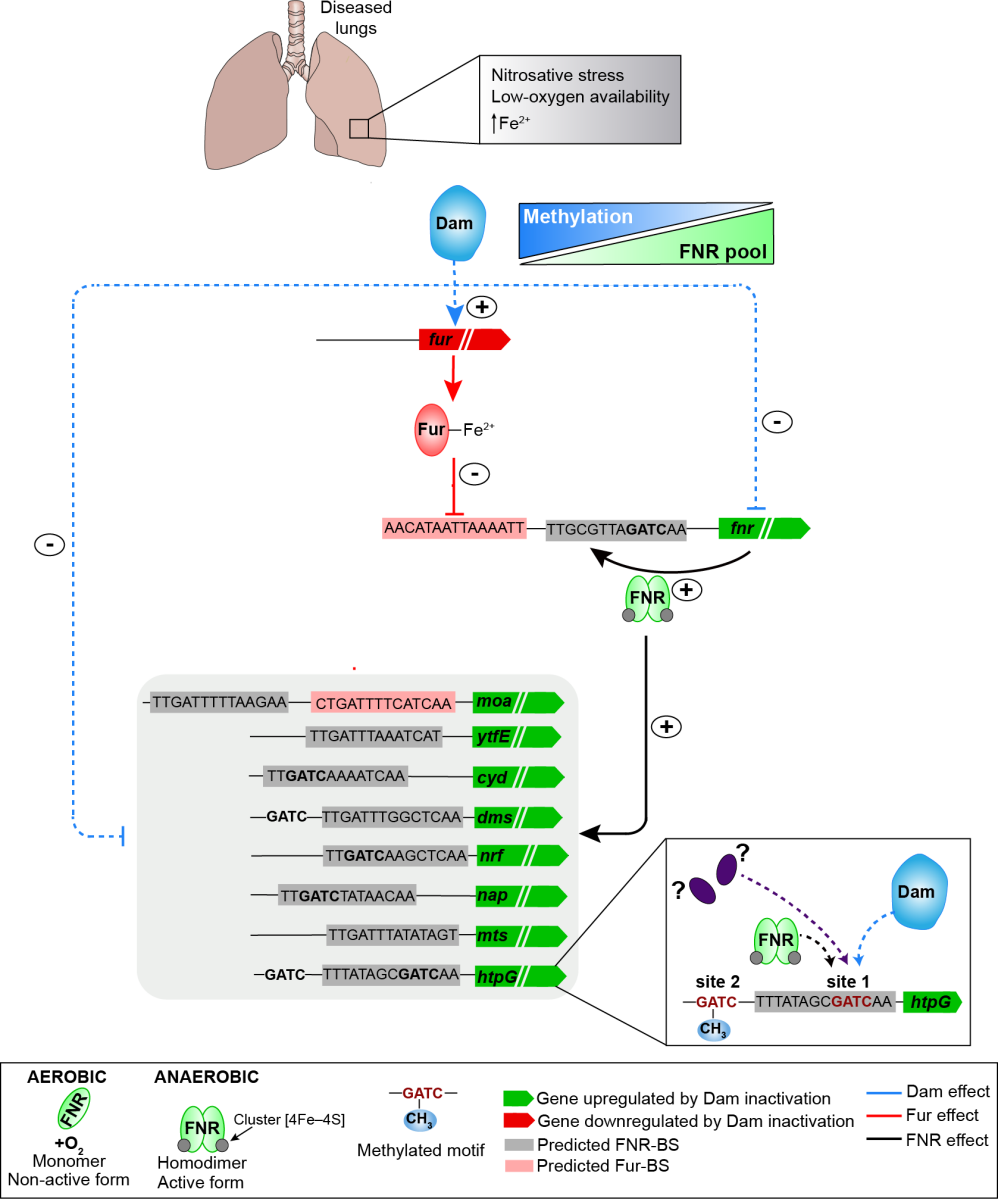
Epigenetic control of Fur and FNR contributes to *H. influenzae* survival in low-oxygen environments during lung infection. Dam methylation positively regulates the expression of the *fur* gene, which in turn represses the expression of *fnr*. Results suggest an inverse correlation between Dam methylation and expression of *fnr* and the FNR regulon. In low-oxygen environments, higher intracellular Fe^2+^ availability may help Fe^+2^-Fur formation and, accordingly, Fur binding and repression of target genes such as *fnr*. FNR, which requires low-oxygen conditions to be active, positively regulates its own expression and the expression of a panel of systems including the molybdenum cofactor biosynthetic cluster, nitrate, nitrite and S-/N-oxide reductases, repair, and transport systems involved in *H. influenzae* anaerobic defense against nitrosative damage produced within the diseased lungs (FNR regulon genes shown inside a light gray box). This work also shows the first case of phenotypic variation in a population of isogenic *H. influenzae* cells controlled by methylation, exemplified by undermethylation in the *htpG* promoter region, which is a gene associated with the bacterial responses to heat stress. A combination of methylation by Dam, methylation blocking by unknown factor(s), and FNR seems to regulate the *htpG* promoter-regulatory region. Downstream effectors of the regulatory network identified in this study are indicated, i.e., the *moa*, *nap*, *dms*, *mtsZ*, *nrf*, *cyd*, *htpG,* and *ytfE* loci. Predicted regulatory regions are shown, including GATC motifs (bold), putative FNR (gray boxes), or Fur (red boxes) binding sites. Proposed epigenetic regulation is indicated with blue dashed arrows; proposed transcriptional factor regulatory events are indicated by red (Fur) or black (FNR) arrows.

Here, we present a multifactorial regulatory network where Dam methylation and the master transcriptional regulators FNR and Fur regulate gene expression in the human pathobiont *H. influenzae* during airway infection. Our use of Tn-seq, SMRT sequencing, and RNA-seq analyses was key to deciphering this network and showed great complementarity, as previously hypothesized (20). In the model shown in Fig. 7, we highlight a repertoire of newly identified elements that make key contributors to regulate the ability of *H. influenzae* to mount a defense to damaging stressors within the airways under low-oxygen conditions: following a putative hierarchical organization, Dam methylation positively regulates the expression of *fur*, which in turn represses *fnr* gene expression. This regulation may be independent of oxygen availability, according to the observed lower *fur* and higher *fnr* gene expression upon Dam inactivation in both aerobic and anaerobic conditions. It should be noted that FNR requires low oxygen to be active as a master regulator to positively regulate its own expression, and the expression of, among others, the nitrate (*nap*), nitrite (*nrf*) and S-/N-oxide reductases (*dms* and *mtsZ*), and repair (*ytfE*) and transport (*cyd*) systems. These systems contribute to the bacterial anaerobic defense against damaging nitrogen reactive species produced by neutrophils, macrophages, and eosinophils within the airways (3). In *E. coli*, levels of active Fur are increased in anaerobiosis because the intracellular Fe^2+^ pool is higher than in aerobiosis. Higher Fe^2+^ availability helps the formation of more Fe^+2^-Fur, and, accordingly, Fur binding and repression increase (14). We can only speculate, but this may also happen in *H. influenzae*. Indeed, anaerobic Fe^+2^-Fur could also contribute to the less pronounced *fnr* upregulation observed in anaerobic than in aerobic samples in this complex regulatory network. Fur repression of multiple FNR-activated operons has been shown in *E. coli* (41), and we present here a case in *H. influenzae*. Of note, FNR and Fur predicted binding sites were also found in the molybdenum cofactor biosynthetic cluster *moaACDE* promoter-regulatory region, and its expression seems to be positively regulated by both regulators (Fig. 3C; Fig. S6), same as observed in *E. coli* (45). Finally, we acknowledge that the uncovered network is incomplete since we did not find GATC motifs linking methylation and *fur* expression in the *fur* promoter-regulatory region. The extent of these observations will be assessed in further work.

Our results showed that the GATC motifs located within the regulatory regions of the *fnr* and *dmsA* loci do not seem to be direct contributors to the observed epigenetic regulation. Also, GATC motifs are embedded within several predicted FNR-BS, but methylation is observed in both the WT and Δ*fnr* strains, suggesting that FNR does not interfere with the methylation of those motifs under the tested conditions. Exception to this is the GATC site 1 of the *htpG* heat-shock chaperone gene promoter region, which also overlaps with a predicted FNR-BS. GATC site 1 undermethylation was observed, regulation of *htpG* gene expression could not be assessed by performing RT-qPCR analyses due to existing heterogeneity (Fig. S7), and required single-cell analyses on a panel of GFP reporter strains. This allowed revealing the first case of phenotypic variation controlled by methylation in *H. influenzae*. Such epigenetic regulation showed modulation by oxygen availability and may involve a combination of methylation by Dam and methylation blocking by other proteins. Also taking into account the existence of two GATC sites, 1 and 2, in the *htpG* promoter-regulatory region, the observed undermethylation of site 1 may lead to changeable methylation patterns having an effect on the FNR regulatory role (Fig. 7, right inset). This complex regulation with Dam methylation, FNR, and currently unknown factors will be studied in further work.

We acknowledge that the results of our multiomic approach may be contingent on growth media and conditions. Here, all assays used sBHI bacterial cultures, and modifying oxygen availability was highly revealing. We focused on the FNR regulon as a whole repertoire of functionally related genes was upregulated upon Dam inactivation. However, other potentially relevant hits were found, such as genes involved in glycerol and glycerol-3P uptake and metabolism, also upregulated in the Δ*dam* strain upon anaerobic growth (Fig. S4C). Glycerol is a product of phosphatidylcholine degradation, which is a major lung surfactant component. It is transported by host cell transmembrane aquaporins whose altered expression correlates with increased mucus production and compromised lung function in COPD (46). Moreover, G3P homeostasis may be key to bacterial fitness as excessive G3P is often toxic (47), highlighting the potential of G3P dehydrogenases as drug targets. Epigenetic regulation of glycerol metabolism will be analyzed in future studies.

Conversely, aiming to expand our observations from reference (RdKW20) to clinical strains, gene inactivation was attempted in strains P602, P615, P621, P665, and P672 but was successful only in P621 and P665. Although limited to these isolates, the effect of *dam* inactivation was similar when compared to RdKW20, confirming some generality in our findings. In contrast, the observed Fur-mediated repression of *fnr* expression requires further discussion, as the Fur regulon was previously studied by Harrison and co-authors, see reference 13, in the *H. influenzae* 86-028NP strain in aerobically grown cultures, and *fnr* was not part of it. Different experimental procedures (RNA-seq versus microarray), genomic background, and experimental conditions were used, which may explain such differences despite sequence conservation in the *fnr* promoter-regulatory region. Also, our results led us to consider that bacterial defense against nitrosative stress may be a functional implication of the proposed regulation. If so, Dam inactivation in low-oxygen conditions may reduce *H. influenzae* sensitivity to NO donors as it upregulates *fnr* gene expression. However, we observed comparable sensitivity to the NO donor, GSNO, by the *dam* and *fnr* mutant strains (Fig. S8), which does not support our hypothesis. This observation highlights Dam's contribution to nitrosative stress defense by unknown mechanisms requiring further work.

Of note, some of the FNR regulon genes shown to be epigenetically regulated (i) are involved in bacterial defense against host-induced S- and N-oxide stress (*dmsA* and *mtsZ* methione sulfoxide reductases [42, 48, 49]); (ii) are also part of *H. influenzae*’s respiratory chain (*napA* nitrate reductase, *dmsA* and *mtsZ* methione sulfoxide reductases, and *nrfA* nitrite reductase); and (iii) previous work showed their upregulation in anaerobiosis (50). *H. influenzae* catabolizes glucose by respiration-assisted fermentation, where the respiratory chain alleviates redox imbalances caused by incomplete glucose oxidation and, at the same time, provides a means of converting a variety of compounds including nitrite and nitrate arising as part of the host defenses (50). Our results suggest that epigenetic mechanisms may also contribute to regulating bacterial metabolism shifts in environments where damage reduces oxygen availability.

Finally, host cell oxygen is a key signal molecule controlling a large number of transcriptional changes mediated by hypoxia-inducible factors. Hypoxia (low-oxygen levels in body tissues) is common in diseased states, and it may be present in pockets of diseased lung tissue. The ventilation/perfusion mismatch that results from progressive airflow limitation and emphysematous destruction of the pulmonary capillary bed is a key driver of hypoxia in COPD. Hypoxia increases in prevalence as disease severity increases and can start a chain reaction that leads to low oxygen in the blood or hypoxemia during COPD exacerbations, with significant drops in arterial oxygen pressure, consequential decreases in oxygen saturation, and a key reason for the shortness of breath in COPD patients (51). Overall, we provide new insights toward understanding selective conditions encountered by *H. influenzae* and the regulatory mechanisms that allow bacteria to survive defensive and pathophysiological features within the diseased lung environment.

## SUPPLEMENTAL MATERIAL

Data Set S1 mbio.01355-25-s0001.xlsx Bacterial genes underrepresented in lung homogenates of mice infected with a transposon mutant library generated in the RdKW20 strain.

Data Set S2 mbio.01355-25-s0002.xlsx Conservation of the *htpG-nif3* intergenic region and DEGs between RdKW20 WT and mutant strains grown in aerobiosis or anaerobiosis.

Supplemental Material mbio.01355-25-s0003.pdf Captions for Data Sets S1 and S2; Supplemental Methods; Tables S1 to S3; Figures S1 to S8.

ASM does not own the copyrights to Supplemental Material that may be linked to, or accessed through, an article. The authors have granted ASM a non-exclusive, world-wide license to publish the Supplemental Material files. Please contact the corresponding author directly for reuse.
