# Supplementary material for: Epigenetic control of the ferric uptake regulator (Fur) and fumarate nitrate reductase (FNR) master regulatory proteins contributes to Haemophilus influenzae survival during lung infection
Source: mBio. 2025 Jul 23;16(8):e01355-25. doi: 10.1128/mbio.01355-25 (PMC12345214; doi:10.1128/mbio.01355-25)
Supplement: Supplemental Material — Captions for Data Sets S1 and S2; Supplemental Methods; Tables S1 to S3; Figures S1 to S8. [file mbio.01355-25-s0003.pdf]

Supplemental Material

Epigenetic control of the ferric uptake regulator (Fur) and fumarate nitrate reductase (FNR) master regulatory proteins contributes to *Haemophilus influenzae* survival during lung infection

Celia Gil-Campillo[1,2], Begoña Euba[1,2], Irene Rodríguez-Arce[1], David San León[3,4], Mary C. Marino[5,6], Javier Asensio-López[1,2,7], Nahikari López-López[1], Joshua C. Mell[5,6], Gabriel Gutiérrez[8], Jeroen D. Langereis[9], María Antonia Sánchez-Romero[10,11#], Junkal Garmendia[1,2,11#]

[1]Instituto de Agrobiotecnología, Consejo Superior de Investigaciones Científicas (IdAB-CSIC)-Gobierno de Navarra, Mutilva, Spain;

[2]Centro de Investigación Biomédica en Red de Enfermedades Respiratorias (CIBERES), Madrid, Spain;

[3]Department of Systems Biology, Centro Nacional de Biotecnología, CSIC, Madrid, Spain;

[4]Interdisciplinary Platform for Sustainable Plastics towards a Circular Economy-Spanish National Research Council (SusPlast-CSIC), Madrid, Spain;

[5]Department of Microbiology and Immunology, College of Medicine, Drexel University, Philadelphia, PA, 19104, USA;

[6]Center for Genomic Sciences, Institute of Molecular Medicine and Infectious Disease, Drexel University, Philadelphia, PA, 19104, USA;

[7]Asociación de la Industria Navarra (AIN)-Gobierno de Navarra, Cordovilla, Spain;

[8]Departamento de Genética, Facultad de Biología, Universidad de Sevilla, Andalucía, Spain;

[9]Department of Laboratory Medicine, Laboratory of Medical Immunology, Radboud Community for Infectious Diseases, Radboud University Medical Center, Nijmegen, the Netherlands;

[10]Departamento de Microbiología y Parasitología, Facultad de Farmacia, Universidad de Sevilla, Andalucía, Spain;

[11]Conexión Antimicrobial Resistance-Spanish National Research Council (AMR-CSIC), Spain

Content:

Dataset S1. Bacterial genes under-represented in lung homogenates of mice infected with a transposon mutant library generated in the RdKW20 strain. Sheet 1, complete list of genes found to be under-represented; sheet 2, list of genes found to be under-represented after infection of mice with normal lung function (N24hpi); sheet 3, list of genes found to be under-represented after infection of mice with lung emphysema (E24hpi); sheet 4, list of genes commonly found to be under-represented after infection of mice with normal lung function and with emphysema.

Dataset S2. Conservation of the *htpG-nif3* intergenic region (sheet 1). DEGs between RdKW20 WT and Δ*dam* strains grown in aerobiosis (sheet 2), or in anaerobiosis (sheet 3). DEGs between RdKW20 WT and Δ*fur* strains grown in anaerobiosis (sheet 4). Green highlights, genes belonging to the FNR regulon over-expressed in Δ*dam* compared to WT. In sheet 3, the *fur* gene is highlighted in red, row 236.

Supplementary methods

Table S1. Bacterial strains used in this study.

Table S2. Plasmids used in this study.

Table S2. Primers used in this study.

Figure S1. Summarized outcome of genome-wide screening of *H. influenzae* genes required for *in vivo* survival.

Figure S2. Growth of *H. influenzae* strains used in this study.

Figure S3. Flow cytometry analysis of *nif3* gene expression.

Figure S4. DEGs commonly found between assay conditions, aerobiosis and anaerobiosis.

Figure S5. Analysis of GATC methylation in the promoter region of the *fnr* gene.

Figure S6. Expression of the *moaA* gene in the WT and Δ*fur* strains by RT-qPCR.

Figure S7. Expression of the *htpG* gene by RT-qPCR.

Figure S8. Survival of *H. influenzae* RdKW20 strains in the presence of the NO donor GNSO.

Supplementary Methods

Bacterial growth. *H. influenzae* strains were grown on PVX agar for 12 h in aerobiosis or anaerobiosis. Depending on the assay, growth was then monitored as follows:

(i) two to five colonies were inoculated in 10 mL sBHI and incubated at 37ºC with 5% CO_2 for 12 h with shaking (90 r.p.m.). Cultures were diluted to OD_{600}=0.05 in sBHI, incubated in sterile 250 mL flasks with 25 mL sBHI and shaking (180 r.p.m.). OD_{600} were recorded every 1 h for up to 8 h.

(ii) (ii) Bacterial suspensions collected from PVX agar growth were normalized to OD_{600}=0.2 in sBHI. Afterwards, 40 µL aliquots were transferred to individual wells in 96-well plates with 160 µL sBHI per well. Plates were incubated at 37ºC for up to 12 h in a Spectro Star Nano (BGM Labtech), and OD_{600} was measured in 1 h intervals. In all 96-well experiments, each growth curve was corrected to its respective blank value (sBHI), and assays were performed in triplicate on at least two independent occasions ($n\geq2$).

Readout transposon mutant libraries. Tn-seq was performed as previously described for *H. influenzae* (1) with some modifications. For NextSeq sequencing, a modified primer (PBGSF23_NextSeq) was used (**Table S3**). In total, 24 adapters with unique barcodes (A through X) were used to multiplex Tn-seq libraries. PCR products were gel-purified and sequenced on an Illumina NextSeq 500 according to the manufacturer's protocol. Genomic DNA was isolated from the recovered mutant libraries with a QIAgen Genomic-tip 20/G (Qiagen). A 200 µL solution with 2 µg of mutant library genomic DNA in CutSmart buffer (New England BioLabs) with 50 µM *S*-adenosylmethionine was digested with 10 U of *Mme*I (New England BioLabs) for 4 h at 37ºC and dephosphorylated with 1 U of calf intestine alkaline phosphatase (Invitrogen) for 30 min at 50°C. The reaction product was extracted with 200 µL phenol-chloroform-isoamyl alcohol (25:24:1), subsequently extracted with 200 µL

chloroform-isoamyl alcohol (24:1), and ethanol precipitated, and the dried DNA pellet was dissolved in 20 µL H_2O. Tn-seq adapters with a 6-bp barcode were prepared by combining 5 nmol of two matching oligonucleotides (**Table S3**) in 1x TE buffer and 50 mM NaCl in a total volume of 50 µL and subjecting the mixture to a 10 min denaturation step at 95ºC and an annealing step in which the reaction mixture was slowly cooled to RT. A 20 µL solution with a 200-pmol adapter was phosphorylated with T4 polynucleotide kinase (3´phosphatase minus) (New England BioLabs) in T4 DNA ligase buffer (New England BioLabs) for 5 min at 37ºC and heat inactivated for 10 min at 70ºC. Ligation of 100 ng of dephosphorylated *Mme*I restriction fragments with 2 pmol of phosphorylated adapter was performed in the presence of T4 DNA ligase buffer with 2 U of T4 DNA ligase (New England BioLabs) in a total volume of 20 µL for 1 h at 16ºC. Immediately after ligation, Tn-seq DNA probes were generated by PCR with 2.5 µL of the ligation reaction mixture as the template, 20 pmol of PBGSF23_NextSeq and PBGSF31 primers, high-fidelity (HF) buffer, 0.2 mM deoxynucleoside triphosphate (dNTP) mix, and 1 U of Phusion DNA polymerase in a total volume of 50 µL. PCR cycling conditions were as follows: 72°C for 1 min and 98°C for 30s, followed by 28 cycles of 98ºC for 30 s, 57°C for 30 s, and 72°C for 10 s, with a final step at 72ºC for 5 min. The resulting PCR product of 130 bp was purified from the PCR with a Minelute Reaction Cleanup Kit (Qiagen). Samples with up to 24 different 6-bp barcodes were pooled and sequenced in a round of 35 cycles on the Illumina Nextseq500.

Tn-seq data analysis. FASTQ files with 35-bp sequences were imported in the web-based interface Essentials (2). Of note, the first nucleotide of the sequence reads often had a poor quality and was omitted. As a result, only the last 5 bp of the 6-bp barcode sequence were available for data analysis. To identify these barcodes, a mismatch of 2 bp was allowed. After removal of the barcode and transposon sequences, the length of the remaining transposon-flanking genomic sequence was set at 17 bp. Alignment of this sequence with the forward strand

of the RdKW20 reference genome should give a match of at least 15 bp. Count data (i.e., pseudoreads) were generated per unique sequence read and per gene. Normalization factors were calculated using the trimmed mean of *M* values (TMM). Pseudoreads in the control and target samples were tested for significant differences ($P<0.001$) by the quantile-adjusted conditional maximum likelihood (qCML) method assuming moderated tag-wise dispersion of replicates. The prior value to determine the amount of smoothing of tag-wise dispersions was set at 10. A Benjamin-Hochberg (B-H) adjusted pvalue (adjusted P of 0.05) was used. Gene essentiality was determined by comparing the expected number of reads per gene (based on the number of insertion sites per gene, the mutant library size, and the sequencing depth) and the measured number of reads per gene. Significantly underrepresented genes were considered essential and omitted from the data analysis. All genes found to be underrepresented *in vivo* compared to sBHI growth were searched in KEGG, Pubmed and Uniprot; in addition, BLASTn was performed using the sequence of each underrepresented gene as query against all *H. influenzae* genomes available at NCBI. Remaining unidentified genes were grouped as hypothetical.

RNA extraction and purification for RNA sequencing (aerobic cultures). Bacterial RNA was isolated using NucleoSpin RNA kit (Macherey-Nagel) as specified by the manufacturer. Briefly, ~$5x10^9$ CFU were pelleted by centrifugation at 14,000 r.p.m. for 5 min, resuspended in 100 µL TE buffer (10 mM Tris-HCl, 1 mM EDTA, pH 8) containing 1 mg/mL lysozyme by vigorous vortexing, and incubated at 37ºC for 10 min. Cells were lysed with 350 µL Buffer RA1 and 3.5 µL β-mercaptoethanol. Lysates were filtered through NucleoSpin Filter units to reduce viscosity, and mixed with 350 µL 70% ethanol. RNA was applied to NucleoSpin RNA columns, salt was removed using membrane desalting buffer (MDB), one on-column rDNase treatment step was included, samples were cleaned with RAW2 and RA3 buffers, and RNA was eluted.

RNA-seq data analysis. Total RNA was isolated as described above and in the main text. The adapters were removed from the sequenced libraries by TrimGalore v0.6.10 (www.bioinformatics.babraham.ac.uk/projects/trim_galore/) using default parameters for paired-end Illumina reads, and reads <20 bps and/or error rate (TrimGalore option "-e") higher than 0.1 were discarded. Previous to the mapping step, the genome was indexed with STAR v2.7.10 (3) with the parameters –runMode genomeGenerate–genomeSAindexNbases 9.4. The remaining reads were mapped to the *H. influenzae* RdKW20 (GCA_000027305.1) genome using STAR using default parameters with the following exceptions: "–outputMultimapperOrder random" and "–twopassMode basic." Before quantification, duplicated reads were marked with Picard tools v3.2 (https://broadinstitute.github.io/picard/). Quantification was done by HTSeq-count included into HTSeq v.2.0.5 (4), using the NCBI annotation in GTF format with the option "–stranded no." Statistical analysis and FPKM calculation were done using DESeq2 v1.44.0 (5) (R package). Expression tracks were generated with the function bamCoverage included into the software deepTools v3.5.2. (6) with the parameter –normalizeUsing RPKM.

H. influenzae differentially expressed genes, pathways or biological functions, with $log_2FC>1$ or $log_2FC<-1$ were searched in KEGG, Pubmed, Uniprot and STRING; in addition, BLASTn was performed using the sequence of each DEG as query against all *H. influenzae* genomes available at NCBI. Remaining unidentified genes were grouped as hypothetical. rRNA genes were discarded during the analysis. STRING analyses of differentially regulated genes were performed and visualized by using Cytoscape 3.7.1 (7, 8).

RT-qPCR. Reverse transcription was performed using 1 μg RNA by PrimerScript RT Reagent kit (Takara). cDNA diluted 1:10 was used as template in a 20 μL reaction mixture containing 1X SYBR Premix Ex Taq II (Tli RNaseH Plus) (Takara), and specific primers pairs for each gene (**Table S3**), designed with Primer3 software. Fluorescence was analyzed with AriaMx

Real-Time PCR System (Agilent Technologies) and QuantStudio 5 real-time PCR System (Thermo Fisher Scientific). The comparative threshold cycle (Ct) method was used to obtain relative quantities of mRNA that were normalized using bacteria *gyrA* gene as endogenous control. Bacterial cultures were grown at least three times, and all samples were processed with technical triplicates ($n \geq 3$). Y axes are labeled as gene expression.

RNS sensitivity assays. Strains were grown at 37ºC in PVX agar for 12 h under anaerobic conditions. Bacterial suspensions collected from PVX agar growth were normalized to OD_{600}=0.4 in sBHI. Afterwards, 1 mL aliquots were transferred to individual tubes with 4 mL of sBHI. Cultures were grown at 37ºC and anaerobic conditions until the exponential phase (OD_{600}=0.3). Afterwards, 200 µL aliquots were transferred to individual wells in 96-well plates with 50 µL of GSNO. Triplicate 200 µL aliquots of each culture were incubated in 96-well plates overnight in an anaerobic chamber in the presence or absence of 50 µL GSNO, and then diluted and plated on sHTM agar for CFU counting. GSNO was used at a final concentration of 0,5 mM. Assays were performed in triplicate on at least two independent occasions ($n \geq 2$).

Table S1. Bacterial strains used in this study.

Strain	Description	Source
E. coli		
TOP10	Cloning strain. F-*mcrA*Δ(*mrr-hsd*RMS-*mcr*BC) Φ80*lac*ZΔM15 Δ*lac*X74 *rec*A1 *ara*D139 Δ(*ara,leu*)7697 *gal*U*gal*K*rps*L (Str^{R}) *end*A1 *nup*G	Thermofisher Scientific
SW102	Derived from DY380; it contains a defective λ prophage with the recombination proteins *exo*, *bet*, and *gam* being controlled by the temperature-sensitive repressor *cI857*	(9)
H. influenzae		
RdKW20	Laboratory strain, capsule-deficient serotype d	(10)
RdKW20Δ*sspA*/P953	*sspA*::*ermC,* Erm^{R}	This study
RdKW20Δ*atpD*/P1011	*atpD*::*ermC,* Erm^{R}	This study
RdKW20Δ*znuA*/P1012	*znuA*::*ermC,* Erm^{R}	This study
RdKW20Δ*relA*/P1503	*relA*::*spec,* Spec^{R}. Strategy C	This study
RdKW20Δ*dam*/P1022	*dam*::*ermC,* Erm^{R}	This study
RdKW20Δ*fnr*/P1221	*fnr*::*spec,* Spec^{R}. Strategy A	This study
RdKW20Δ*fnr*/P1220	*fnr*::*ermC,* Erm^{R}	This study
RdKW20Δ*fur*/P583	*fur*::*spec,* Spec^{R}. Strategy B	This study
RdKW20Δ*dam*Δ*fnr/*P1231	*dam*::*ermC,* Erm^{R}/*fnr*::*spec,* Spec^{R}. Strategy A	This study
P595-8370	NTHi isolate from COPD patient 2, CT 14	(11)
P602-8883	NTHi isolate from COPD patient 4, CT 48	(11)
P615-8618	NTHi isolate from COPD patient 6, CT 48	(11)
P617-9224	NTHi isolate from COPD patient 6, CT 44	(11)
P621-7028	NTHi isolate from COPD patient 7, CT 48	(11)
P636-8296	NTHi isolate from COPD patient 9, CT 44	(11)
P641-4342	NTHi isolate from COPD patient 10, CT 18	(11)
P642-4396	NTHi isolate from COPD patient 10, CT 18	(11)
P650-8603	NTHi isolate from COPD patient 10, CT 14	(11)
P652-8881	NTHi isolate from COPD patient 10, CT 73	(11)
P657-8759	NTHi isolate from COPD patient 11, CT 100	(11)
P662-7189	NTHi isolate from COPD patient 12, CT 106	(11)
P665-7858	NTHi isolate from COPD patient 12, CT 76	(11)
P669-6977	NTHi isolate from COPD patient 13, CT 3	(11)
P672-7661	NTHi isolate from COPD patient 13, CT 7	(11)
P676-2514	NTHi isolate from COPD patient 3, CT 16	(11)
P679-2791	NTHi isolate from COPD patient 3, CT 16	(11)
P621Δ*dam/*P1023	*dam*::*ermC,* Erm^{R}	This study
P665Δ*dam/*P1024	*dam*::*ermC,* Erm^{R}	This study
RdKW20-*htpG*::*gfp*/P1158	RdKW20 derivative, containing a *htpG*::*gfp* transcriptional fusion integrated as a single copy into the chromosome; Spec^{R}	This study
RdKW20Δ*dam*-*htpG*::*gfp*/P1160	RdKW20Δ*dam* derivative, containing a *htpG*::*gfp* transcriptional fusion integrated as a single copy into the chromosome; Erm^{R}, Spec^{R}	This study
RdKW20Δ*fnr*-*htpG*::*gfp*/P1232	RdKW20Δ*fnr* derivative, containing a *htpG*::*gfp* transcriptional fusion integrated as a single copy into the chromosome; Erm^{R}, Spec^{R}	This study
RdKW20-*nif3*::*gfp*/P1159	RdKW20 derivative, containing a *nif3*::*gfp* transcriptional fusion integrated as single copy into the chromosome; Spec^{R}	This study
RdKW20Δ*dam*-*nif3*::*gfp*/P1161	RdKW20Δ*dam* derivative, containing a *nif3*::*gfp* transcriptional fusion integrated as single copy into the chromosome; Erm^{R}, Spec^{R}	This study
P621-*htpG*::*gfp*/P1163	P621 derivative, containing a *htpG*::*gfp* transcriptional fusion integrated as a single copy into the chromosome; Spec^{R}	This study

P621Δ*dam-htpG*::*gfp*/P1164	P621Δ*dam* derivative, containing a *htpG*::*gfp* transcriptional fusion integrated as a single copy into the chromosome; $Spec^R$	This study
P665-*htpG*::*gfp*/P1187	P665 derivative, containing a *htpG*::*gfp* transcriptional fusion integrated as a single copy into the chromosome; $Spec^R$	This study
P665Δ*dam-htpG*::*gfp*/P1188	P665Δ*dam* derivative, containing a *htpG*::*gfp* transcriptional fusion integrated as a single copy into the chromosome; $Spec^R$	This study
RdKW20WT-*dmsA*-WT/P1476	RdKW20WT isogenic strain; used as WT control strain	This study
RdKW20Δ*dam-dmsA*-WT/P1479	RdKW20Δ*dam* isogenic strain; used as Δ*dam* control strain	This study
RdKW20WT-*dmsA*-GCTC/ P1477	P1476 derivate, containing a GATC to GCTC chromosomal exchange in the *dmsA* gene upstream intergenic region	This study
RdKW20WT-*fnr*WT/P1480	RdKW20 WT isogenic strain- used as WT control strain in Figure 5	This study
RdKW20Δ*dam-fnr*WT/P1481	RdKW20Δ*dam* isogenic strain- used as Δ*dam* control strain in Figure 5	This study
RdKW20WT-*fnr*GCTC/P1508	P1480 derivate containing a GATC to GCTC chromosomal exchange in the *fnr* gene upstream intergenic region	This study
RdKW20WT-*fnr*BS*/P1507	P1480 derivate, containing a TTGCGTTAGATCAA to GGTATGGAGATCAA chromosomal exchange in the *fnr* gene upstream intergenic region	This study
RdKW20Δ*dam-fnr*BS*/P1509	P1481 derivate, containing a TTGCGTTAGATCAA to GGTATGGAGATCAA chromosomal exchange in the *fnr* gene upstream intergenic region	This study
RdKW20WT-*fur*BS*/P1567	P1480 derivate, containing a AACATAATTAAAATT to CCACGCCGGCCCCGG chromosomal exchange in the *fnr* gene upstream intergenic region	This study
RdKW20WT-*htpG*-WT::*gfp*/P1475	P1158 isogenic strain- used as WT control strain; Erm^R, $Spec^R$	This study
RdKW20WT-*htpG*-AATC::*gfp*/P1400	P1158 derivative, containing a *htpG*::*gfp* transcriptional fusion integrated as a single copy into the chromosome, and a TTTATAGCGATCAA to TTTATAGCAATCAA chromosomal exchange in the *htpG* gene upstream intergenic region; Erm^R, $Spec^R$	This study

Table S2. Plasmids used in this study.

Pasmid	Description	Source
pJET1.2/blunt	Cloning vector	Life Technologies
pBSLerm	Plasmid containing an Erm resistance cassette (Erm^R)	(12)
pRSM2832	pKD13 derivative carrying a cassette containing a Spec resistance gene flanked by FRT sites	(9)
pJET1.2-*sspA*	pJET1.2 derivative containing a 2,639 bp DNA fragment carrying the *sspA*RdKW20 gene (639 bp) and its upstream (1,000 bp) and downstream (1,000 bp) flanking regions. Strain P947	This study
pJET1.2-*sspA*::*ermC*	pJET1.2-*sspA* derivative containing a 3,299 bp DNA fragment carrying a *sspA::ermC* disruption cassette. Strain P950	This study
pJET1.2-*atpD*	pJET1.2 derivative containing a 3,277 bp DNA fragment carrying the *atpD*RdKW20 gene (1,374 bp) and its upstream (914 bp) and downstream (989 bp) flanking regions. Strain P1001	This study
pJET1.2-*atpD*::*ermC*	pJET1.2-*atpD* derivative containing a 3,121 bp DNA fragment carrying a *atpD::ermC* disruption cassette. Strain P1008	This study
pJET1.2-*znuA*	pJET1.2 derivative containing a 2,934 bp DNA fragment carrying the *znuA*RdKW20 gene (1,014 bp) and its upstream (958 bp) and downstream (962 bp) flanking regions. Strain P982	This study
pJET1.2-*znuA*::*ermC*	pJET1.2-*znuA* derivative containing a 3,219 bp DNA fragment carrying a *znuA::ermC* disruption cassette. Strain P983	This study
pR412	Plasmid containing a $Spec^R$ cassette	(13)
pJET1.2-*dam*$_{RdKW20}$	pJET1.2 derivative containing a 2,473 bp DNA fragment carrying the *dam*$_{RdKW20}$ gene (861 bp) and its upstream (761 bp) and downstream (851 bp) flanking regions. Strain P1005	This study
pJET1.2-*dam*$_{P621}$	pJET1.2 derivative containing a 2,473 bp DNA fragment carrying the *dam*$_{P621}$ gene (861 bp) and its upstream (761 bp) and downstream (851 bp) flanking regions. Strain P1006	This study
pJET1.2-*dam*$_{P665}$	pJET1.2 derivative containing a 2,473 bp DNA fragment carrying the *dam*$_{P665}$ gene (861 bp) and its upstream (761 bp) and downstream (851 bp) flanking regions. Strain P1007	This study
pJET1.2-*dam*$_{RdKW20}$::*ermC*	pJET1.2-*dam* derivative containing a 2,945 bp DNA fragment carrying a *dam*$_{RdKW20}$*::ermC* disruption cassette. Strain P1013	This study
pJET1.2-*dam*$_{P621}$::*ermC*	pJET1.2-*dam* derivative containing a 2,945 bp DNA fragment carrying a *dam*$_{P621}$*::ermC* disruption cassette. Strain P1014	This study
pJET1.2-*dam*$_{P665}$::*ermC*	pJET1.2-*dam* derivative containing a 2,945 bp DNA fragment carrying a *dam*$_{P665}$*::ermC* disruption cassette. Strain P1015	This study
pJET1.2-*fnr*	pJET1.2 derivative containing a 2,278 bp DNA fragment carrying the *fnr* gene (774 bp) and its upstream (513 bp) and downstream (991 bp) flanking regions. Strain P1211	This study
pJET1.2-*fnr*::*Spec*	pJET1.2-*fnr* derivative containing a 2,837 bp DNA fragment carrying a *fnr::spec* disruption cassette. Strain P1213	This study
pJET1.2-*fnr*::*ermC*	pJET1.2-*fnr* derivative containing a 2,839 bp DNA fragment carrying a *fnr::ermC* disruption cassette. Strain P1216	This study
pJET1.2-*fur*	pJET1.2 derivative containing a 2,441 bp DNA fragment carrying the *fur* gene (441 bp) and its upstream (1,000 bp) and downstream (1,000 bp) flanking regions.	This study
pJET*fur*::Spec	pJET1.2-*fur* derivative containing a 4,091 bp DNA fragment carrying a *fur::spec* disruption cassette. Strain P587	This study
pJET-*nif3*RdKW20+*down*	pJET1.2 derivative containing a 1,348 bp DNA fragment carrying part of the *nif3* gene (from base 72 to 756) and its downstream (663 bp) region (template used, genomic DNA from strain RdKW20). Strain P1130	This study
pJET-*nif3*P621+*down*	pJET1.2 derivative containing a 1,348 bp DNA fragment carrying part of the *nif3* gene (from base 72 to 756) and its downstream (663 bp) region (template used, genomic DNA from strain P621). Strain P1131	This study

pJET-*nif3*P665+*down*	pJET1.2 derivative containing a 1,348 bp DNA fragment carrying part of the *nif3* gene (from base 72 to 756) and its downstream (663 bp) region. (template used, genomic DNA from strain P665). Strain P1132	This study
pJET-*htpG*RdKW20 +*down*	pJET1.2 derivative containing a 1,339 bp DNA fragment carrying part of the *htpG* gene (from base 1,329 to 1,896) and its downstream (771 bp) region (template used, genomic DNA from strain RdKW20). Strain P1133	This study
pJET-*htpG*P621+*down*	pJET1.2 derivative containing a 1,339 bp DNA fragment carrying part of the *htpG* gene (from base 1,329 to 1,896) and its downstream (771 bp) region (template used, genomic DNA from strain P621). Strain P1134	This study
pJET-*htpG*P665+*down*	pJET1.2 derivative containing a 1,339 bp DNA fragment carrying part of the *htpG* gene (from base 1,329 to 1,896) and its downstream (771 bp) region (template used, genomic DNA from strain P665). Strain P1135	This study
pJET 1.2.-*nif3*$_{RdKW20}$*::gfp*	pJET-*nif3* derivative containing a 3,609 bp DNA fragment carrying a *nif3*$_{RdKW20}$*::gfp,* transcriptional fusion. Strain P1152	This study
pJET 1.2.-*nif3*$_{P621}$*::gfp*	pJET-*nif3* derivative containing a 3,609 bp DNA fragment carrying a *nif3*$_{P621}$*::gfp,* transcriptional fusion. Strain P1153	This study
pJET 1.2.-*nif3*$_{P665}$*::gfp*	pJET-*nif3* derivative containing a 3,609 bp DNA fragment carrying a *nif3*$_{P621}$*::gfp,* transcriptional fusion. Strain P1154	This study
pJET 1.2.-*htpG*RdKW20*::gfp*	pJET-*htpG* derivative containing a 3,599 bp DNA fragment carrying a *htpG*$_{RdKW20}$*::gfp* transcriptional fusion. Strain P1155	This study
pJET 1.2.-*htpG*P621*::gfp*	pJET-*htpG* derivative containing a 3,599 bp DNA fragment carrying a *htpG*$_{P621}$*::gfp* transcriptional fusion. Strain P1156	This study
pJET 1.2.-*htpG*P665*::gfp*	pJET-*htpG* derivative containing a 3,599 bp DNA fragment carrying a *htpG*$_{P665}$*::gfp* transcriptional fusion. Strain P1157	This study
pZEP07::Spec^{R}	pZEP07 derivative, Spec^{R}. Strain P1150	This study
pUC18-*dmsA*-WT	pUC18 derivative containing a 2,958 bp DNA fragment carrying: bases 210 to 972 of HI_1028; Spec^{R} gene; bases 1 to 209 of Hi_1048; WT intergenic region (253 bp); bases 1 to 547 of the *dmsA* gene. Strain P1421. Used to generate strains RdKW20 WT-*dmsA*-WT/P1476 and RdKW20Δ*dam-dmsA*-WT/P1479	This study
pUC18-*dmsA*-GCTC	pUC18 derivative containing a 2,958 bp DNA fragment carrying: bases 210 to 972 of HI_1028; Spec^{R} gene; bases 1 to 209 of Hi_1048; intergenic region (253 bp) with GATC to GCTC exchange; bases 1 to 547 of the *dmsA* gene. Strain P1420. Used to generate strain RdKW20WT-*dmsA*-GCTC/ P1477	This study
pUC18-*fnr*WT	pUC18 derivative containing a 3,285 bp DNA fragment carrying: bases 259 to 900 of HI_1424; Spec^{R} gene; bases 1 to 258 of Hi_1424; WT intergenic region (511 bp); bases 1 to 688 of the *fnr* gene. Strain P1478. Used to generate strain RdKW20 WT-*fnr*WT/P1480 and Δ*dam-fnr*WT/P1484	This study
pUC18-*fnr*GCTC	pUC18 derivative containing a 3,285 bp DNA fragment carrying: bases 259 to 900 of HI_1424; Spec^{R} gene; bases 1 to 258 of Hi_1424; intergenic region (511 bp) with GATC to GCTC exchange; bases 1 to 688 of the *fnr* gene. Strain P1487. Used to generate strain RdKW20WT-*fnr*GCTC/P1508	This study
pUC18-*fnr*BS*	pUC18 derivative containing a 3,285 bp DNA fragment carrying: bases 259 to 900 of HI_1424; Spec^{R} gene; bases 1 to 258 of Hi_1424; intergenic region (511 bp) with TTGCGTTAGATCAA to GGTATGGAGATCAA modification of the putative FNR-BS; bases 1 to 688 of the *fnr* gene. Strain P1486. Used to generate strain RdKW20 WT-*fnr*BS*/P1507 and Δ*dam-fnr*BS*/P1509	This study
pUC18-*fur*BS*	pUC18 derivative containing a 3,285 bp DNA fragment carrying: bases 259 to 900 of HI_1424; Spec^{R} gene; bases 1 to 258 of Hi_1424; intergenic region (511 bp) with AACATAATTAAAATT to CCACGCCGGCCCCGG modification of the putative Fur-BS;	This study

	bases 1 to 688 of the *fnr* gene. Strain P1543. Used to generate strain RdKW20WT-*fur*BS*/P1567	
pUC18 *htpG*-WT	pUC18 derivative containing a 3,000 bp DNA fragment carrying: bases 169 to 756 of *nif3*; ErmC^{R} gene; bases 1 to 168 of *nif3*; WT intergenic region (300 bp); bases 1 to 726 of the *htpG* gene. Strain P1423. Used to generate strain RdKW20WT-*htpG*-WT::*gfp*/P1475	This study
pUC18 *htpG*-GATC1	pUC18 derivative containing a 3,000 bp DNA fragment carrying: bases 169 to 756 of *nif3*; ErmC^{R} gene; bases 1 to 168 of *nif3*; WT intergenic region (300 bp) with a modification of GATC site 1 to AATC; bases 1 to 726 of the *htpG* gene. Strain P1386. Used to generate strain RdKW20WT-*htpG*-AATC::*gfp*/P1400	This study

Table S3. Primers used in this study.

Primer name	Primer sequence (5'-3')	Purpose	Source
PBGSF29 ATCACG	TTCCCTACACGACGCTCTTCCGATCTATCACGNN	Tn-seq	(14)
PBGSF30 ATCACG	CGTGATAGATCGGAAGAGCGTCGTGTAGGGAAAGAGT	Tn-seq	(14)
PBGSF29 CGATGT	TTCCCTACACGACGCTCTTCCGATCTCGATGTNN	Tn-seq	(14)
PBGSF30 CGATGT	ACATCGAGATCGGAAGAGCGTCGTGTAGGGAAAGAGT	Tn-seq	(14)
PBGSF29 TTAGGC	TTCCCTACACGACGCTCTTCCGATCTTTAGGCNN	Tn-seq	(14)
PBGSF30 TTAGGC	GCCTAAAGATCGGAAGAGCGTCGTGTAGGGAAAGAGT	Tn-seq	(14)
PBGSF29 TGACCA	TTCCCTACACGACGCTCTTCCGATCTTGACCANN	Tn-seq	(14)
PBGSF30 TGACCA	TGGTCAAGATCGGAAGAGCGTCGTGTAGGGAAAGAGT	Tn-seq	(14)
PBGSF29 ACAGTG	TTCCCTACACGACGCTCTTCCGATCTACAGTGNN	Tn-seq	(14)
PBGSF30 ACAGTG	CACTGTAGATCGGAAGAGCGTCGTGTAGGGAAAGAGT	Tn-seq	(14)
PBGSF29 GCCAAT	TTCCCTACACGACGCTCTTCCGATCTGCCAATNN	Tn-seq	(14)
PBGSF30 GCCAAT	ATTGGCAGATCGGAAGAGCGTCGTGTAGGGAAAGAGT	Tn-seq	(14)
PBGSF29 CAGATC	TTCCCTACACGACGCTCTTCCGATCTCAGATCNN	Tn-seq	(14)
PBGSF30 CAGATC	GATCTGAGATCGGAAGAGCGTCGTGTAGGGAAAGAGT	Tn-seq	(14)
PBGSF29 ACTTGA	TTCCCTACACGACGCTCTTCCGATCTACTTGANN	Tn-seq	(14)
PBGSF30 ACTTGA	TCAAGTAGATCGGAAGAGCGTCGTGTAGGGAAAGAGT	Tn-seq	(14)
PBGSF29 GATCAG	TTCCCTACACGACGCTCTTCCGATCTGATCAGNN	Tn-seq	(14)
PBGSF30 GATCAG	CTGATCAGATCGGAAGAGCGTCGTGTAGGGAAAGAGT	Tn-seq	(14)
PBGSF29 TAGCTT	TTCCCTACACGACGCTCTTCCGATCTTAGCTTNN	Tn-seq	(14)
PBGSF30 TAGCTT	AAGCTAAGATCGGAAGAGCGTCGTGTAGGGAAAGAGT	Tn-seq	(14)
PBGSF29 GGCTAC	TTCCCTACACGACGCTCTTCCGATCTGGCTACNN	Tn-seq	(14)
PBGSF30 GGCTAC	GTAGCCAGATCGGAAGAGCGTCGTGTAGGGAAAGAGT	Tn-seq	(14)
PBGSF29 CTTGTA	TTCCCTACACGACGCTCTTCCGATCTCTTGTANN	Tn-seq	(14)
PBGSF30 CTTGTA	TACAAGAGATCGGAAGAGCGTCGTGTAGGGAAAGAGT	Tn-seq	(14)
PBGSF29 AGTCAA	TTCCCTACACGACGCTCTTCCGATCTAGTCAANN	Tn-seq	This study
PBGSF30 AGTCAA	TTGACTAGATCGGAAGAGCGTCGTGTAGGGAAAGAGT	Tn-seq	This study
PBGSF29 AGTTCC	TTCCCTACACGACGCTCTTCCGATCTAGTTCCNN	Tn-seq	This study
PBGSF30 AGTTCC	GGAACTAGATCGGAAGAGCGTCGTGTAGGGAAAGAGT	Tn-seq	This study
PBGSF29 ATGTCA	TTCCCTACACGACGCTCTTCCGATCTATGTCANN	Tn-seq	This study
PBGSF30 ATGTCA	TGACATAGATCGGAAGAGCGTCGTGTAGGGAAAGAGT	Tn-seq	This study
PBGSF29 CCGTCC	TTCCCTACACGACGCTCTTCCGATCTCCGTCCNN	Tn-seq	This study
PBGSF30 CCGTCC	GGACGGAGATCGGAAGAGCGTCGTGTAGGGAAAGAGT	Tn-seq	This study

PBGSF29 GTCCGC	TTCCCTACACGACGCTCTTCCGATCTGTCCGCNN	Tn-seq	This study
PBGSF30 GTCCGC	GCGGACAGATCGGAAGAGCGTCGTGTAGGGAAAGAGT	Tn-seq	This study
PBGSF29 GTGAAA	TTCCCTACACGACGCTCTTCCGATCTGTGAAANN	Tn-seq	This study
PBGSF30 GTGAAA	TTTCACAGATCGGAAGAGCGTCGTGTAGGGAAAGAGT	Tn-seq	This study
PBGSF29 GTGGCC	TTCCCTACACGACGCTCTTCCGATCTGTGGCCNN	Tn-seq	This study
PBGSF30 GTGGCC	GGCCACAGATCGGAAGAGCGTCGTGTAGGGAAAGAGT	Tn-seq	This study
PBGSF29 GTTTCG	TTCCCTACACGACGCTCTTCCGATCTGTTTCGNN	Tn-seq	This study
PBGSF30 GTTTCG	CGAAACAGATCGGAAGAGCGTCGTGTAGGGAAAGAGT	Tn-seq	This study
PBGSF29 CGTACG	TTCCCTACACGACGCTCTTCCGATCTCGTACGNN	Tn-seq	This study
PBGSF30 CGTACG	CGTACGAGATCGGAAGAGCGTCGTGTAGGGAAAGAGT	Tn-seq	This study
PBGSF29 GAGTGG	TTCCCTACACGACGCTCTTCCGATCTGAGTGGNN	Tn-seq	This study
PBGSF30 GAGTGG	CCACTCAGATCGGAAGAGCGTCGTGTAGGGAAAGAGT	Tn-seq	This study
PBGSF29 ACTGAT	TTCCCTACACGACGCTCTTCCGATCTACTGATNN	Tn-seq	This study
PBGSF30 ACTGAT	ATCAGTAGATCGGAAGAGCGTCGTGTAGGGAAAGAGT	Tn-seq	This study
PBGSF29 ATTCCT	TTCCCTACACGACGCTCTTCCGATCTATTCCTNN	Tn-seq	This study
PBGSF30 ATTCCT	AGGAATAGATCGGAAGAGCGTCGTGTAGGGAAAGAGT	Tn-seq	This study
PBGSF23_NextSeq	CAAGCAGAAGACGGCATACGAGATAGACCGGGGACTT ATCATCCAACCTGT	Tn-seq	This study
PBGSF31	AATGATACGGCGACCACCGAGATCTACACTCTTTCCCT ACACGACGCTCTTCCGATCT	Tn-seq	(13)
sspA-F1/1725	AAACCAGAAACGGTTAAACGCGAC	Disruption cassette	This study
sspA-R1 /1726	AATGAATGCCGTCAAATGCTCTAT	Disruption cassette	This study
sspA-F2/1727	AAAGCGATTAAAGCTTATATGGAA	Disruption cassette	This study
sspA-R2/1728	ACCTGATGGCAATAAATATCATCT	Disruption cassette	This study
atpD -F1/1871	TAGTGCTTGGTAGTAACGGAGAGATAA	Disruption cassette	This study
atpD -R1/1872	ATTAGGATTATTAGAATGTATTGAAGA	Disruption cassette	This study
atpD -F2/1873	CCATATTCCTGAACAAGCGTTCTATAT	Disruption cassette	This study
atpD -R2/1874	CTTGTGGAAATTCAACGTCAATCACCG	Disruption cassette	This study
znuA-F1/1797	TATTACGGTAATGGCTAGAATAGATAA	Disruption cassette	This study
znuA-R1/1798	GCAGTGCTTGAACCTCGTTCTAACACA	Disruption cassette	This study
znuA-F2/1803	GCAACATTCTTGCAATCTACTGCAGAT	Disruption cassette	This study
znuA-R2/1804	TCGGCATTCGCCATCATTGGCGCACTT	Disruption cassette	This study
dam-F1 / 1899	TGGCTTTGCTGCGGCAAGTTATCAGCG	Disruption cassette	This study
dam-R1 / 1900	CAAAATCTAAACCAGCAGTGACGTTA	Disruption cassette	This study
dam-F2 / 1901	CGGGTAAAAGTCCAACGTTCTATTAGC	Disruption cassette	This study

dam-R2 / 1902	TAAAAATGGACGGTGTTTTAACTTAGG	Disruption cassette	This study
SpecC_F	GGATCCCCCGTTTGATTTTTAATGGTAATG	Disruption cassette	This study
SpecC_R	CGAATTGACGCGGAATGGATCC	Disruption cassette	This study
relA_flankA_F	TATGATTTGTGGCGATGCTT	Disruption cassette	This study
relA_flankA_SpecC_R	TGATTAGTACCTATTTTATCACATTACCATTAAAAATCAAACGGGGGATCCTAAGCAATGTTTCCGCATCC	Disruption cassette	This study
relA_flankB_SpecC_F	TAACAGATTAAAAAAATTATAAAAAAATTGGATCCATTCCGCGTCAATTCGCTATTCACCGAGCGGATTGT	Disruption cassette	This study
relA_flankB_R	TGATTGTCATACCTGAAATACCA	Disruption cassette	This study
val_relA_flankA_F	AGCAGTTCGTAGTTATCTTGGAG	Validation	This study
val_SpecC_internal_R	GTCAATGGTTCAGATACGACGAC	Validation	This study
val_SpecC_internal_F	GTTCAAACCATTACGGCTGA	Validation	This study
val_relA_flankB_R	ATCGCATCCTCGTTTTCTTC	Validation	This study
fnr-F1 / 2235	ATAAAAATACTTAAAAAATGGCAATAT	Disruption cassette	This study
fnr-R1 / 2236	CATGTTCTGCAGTATTGCCTAATAATG	Disruption cassette	This study
fnr-F2 / 2237	CAAGGCAAATATATTACAATCAATGAT	Disruption cassette	This study
fnr-R2 / 2238	ATGAATCGCACAACCGCCTGATTGAAT	Disruption cassette	This study
fur-F1 / 1173	CGTCAGCAAGCCAAACCGATTTTA	Disruption cassette	This study
fur-R1 / 1174	AACTTTATCGTCACGTAGGAGCTG	Disruption cassette	This study
fur-casset-F / 1201	CTTAGCTGAATTGGCTTAGTATGTTGTGCATAATAGGAAGAAAAATTATGATTCCGGGGATCCGTCGACC	Disruption cassette	This study
fur-casset-R / 1202	AGGCGACATTTTGTCGCCTTAGATATTATTTATTTTGAATTGTTTTCGTCTGTAGGCTGGAGCTGCTTCG	Disruption cassette	This study
GFP-F1 / 2118	TAAGAAGGAGATATACATATGAG	GFP fusion	This study
Spec_R4 / 2181	AGGCCGCTAAAGCGGCTAAAAGCGCTA	GFP fusion	This study
FragA-*nif3*-F1 / 2104	CAACGGCTTACAAGTCGAAGGTAAAAC	GFP fusion	This study
FragA-*nif3*-RdKW20-R1 / 2105	TTTTGTATTTTATGCTGGATTATCTAT	GFP fusion	This study
FragA-*nif3*-P621-R1/2106	TTTTGTGTTTTATGCTGGATTATCTA	GFP fusion	This study
FragA-*nif3*-P665-R1(2107	TTTTGTATTTTACGCTGGATTATCTAT	GFP fusion	This study
FragB-*nif3*-F1 / 2108	GTGCGGTAAAAATATTTCTTTTTTAAC	GFP fusion	This study
FragB-*nif3*-R1 / 2109	GATTCGTCAAATCACAGGTAAACCAAT	GFP fusion	This study
FragA-*htpG*-F1 / 2110	GGATAGTTATGTTGCAGCGAAAAATAG	GFP fusion	This study
FragA-*htpG*-R1 / 2111	TCAAATGAATTAACCTAACAACTTATT	GFP fusion	This study
FragB-*htpG*-RdKW20-P621-F1 / 2112	GATGAAGTAGCATAAGGGTTAAAATCC	GFP fusion	This study
FragB-*htpG*-P665-F1/2113	GATGAAGTAGTATAAGGGTTAAAATCC	GFP fusion	This study

FragB-*htpG*-R1 / 2114	TGCTGCAAGGGAGCCTTTCATATCCGC	GFP fusion	This study
F1-chequeo-FNR-2 / 2448	TGCAATAATTAAAATGCACAACAT	Confirmation of *fnr* promoter modification	This study
R1-chequeo-FNR-2 / 2449	GACTAATACTGCAATCCTGACAAT	Confirmation of *fnr* promoter modification	This study
F1-chequeo-*htpG*-2/2450	GATTTCTGTTTTACCTTCGACTTG	Confirmation of *htpG* promoter modification	This study
R1-chequeo-*htpG*-2/2451	GAAAGATTTCAAGAGGAATTTAAG	Confirmation of *htpG* promoter modification	This study
F1-chequeo-*dmsA*2/2521	AACTAAATCATAGGTTTGCGTGAA	Confirmation of *dmsA* promoter modification	This study
R1-chequeo-*dmsA*2/2522	AAACTTTGGAATAACAAATTGACT	Confirmation of *dmsA* promoter modification	This study
*dam*2-qPCR-F / 1969	TCCTTTCGCACTAGCCATTT	qPCR	This study
*dam*2-qPCR-R / 1970	TTGATTTCTGTTTTACCTTCGACTT	qPCR	This study
F2-*dam*1-qPCR / 2059	CCGCGAGTTTCTTGATTTTG	qPCR	This study
R2-*dam*1-qPCR / 2060	AGTGGGGGAATGCACTAGC	qPCR	This study
GATC_*fnr*_F1_qPCR / 2225	GCTGCCATAATGCTTTACTCTTCT	qPCR	This study
GATC_*fnr*_R1_qPCR / 2226	GCACAACCGCCTGATTGAAT	qPCR	This study
GATC_*dmsA*_F1_qPCR / 2284	TCGTAAAGATGGGCGTCTG	qPCR	This study
GATC_*dmsA*_R1_qPCR / 2285	CGTTAGCATAGTGGCGTGTTAG	qPCR	This study
F1-*nap*-GATC-qPCR / 2586	AGCCATCGTGAAACTGTGAA	qPCR	This study
R1-*nap*-GATC-qPCR / 2587	GAATGAATGAAAACAACCCAAAA	qPCR	This study
R1-*nrfA*-GATC-qPCR / 2589	TTTCATTCAAGCGTAAGATACCAA	qPCR	This study
F2-*nrfA*-GATC-qPCR / 2590	GCGTGGAACAAGTACAACAGG	qPCR	This study
*gyrA*_F1 / 1078	ATATGTTGGTTGATGGGCAAGG	qRT-PCR	(11)
*gyrA*_R1 / 1079	GGCGAGAAATTGACGGTTTCT	qRT-PCR	(11)
*fur*qPCR-F / 1410	GCGCATATTGTAATCCGTCA	qRT-PCR	This study
*fur*qPCR-R / 1411	TGATTTCACGCTGAGGTTGT	qRT-PCR	This study
htgG-qPCR-F / 1963	CAGTGGAAATGCTGACGAAA	qRT-PCR	This study
htgG-qPCR-R / 1964	CCCAAGTCACAGGATCAACA	qRT-PCR	This study
*fnr*_F1_qPCR / 2205	CAGAAGAACGTTTGGCTGCT	qRT-PCR	This study
*fnr*_R1_qPCR / 2206	CCTTGTACGCTAATCACGCC	qRT-PCR	This study
*cydD*_F1_qPCR / 2288	AAGTGCGGTGGATTTTGAAG	qRT-PCR	This study
*cydD*_R1_qPCR / 2289	AAGCCCATCAACAACGAAAC	qRT-PCR	This study

*ytfE*_F2_qPCR / 2311	TCGCTGTTTCAATTCCAGGTG	qRT-PCR	This study
*ytfE*_R2_qPCR / 2312	ATACAGAACCTCCGCAGCAA	qRT-PCR	This study
F1_*dmsA*_qPCR / 2317	GATTATACCTATGGTGGTGGTTGG	qRT-PCR	This study
R1_*dmsA*_qPCR / 2318	ATCATTTTGGCATTGGAACG	qRT-PCR	This study
nrfA-F1-qPCR / 2545	CCCTTGGGATAATGGTCAAA	qRT-PCR	This study
nrfA-R1-qPCR / 2546	GTGCTGTCAAATGCCTCAAA	qRT-PCR	This study
napH-F1-qPCR / 2547	GGCTGCAAGACATTTACCAGA	qRT-PCR	This study
napH-R1-qPCR / 2548	TAACCCACTCCCAAAGCAAC	qRT-PCR	This study
moaE-F1-qPCR / 2549	ATTCATCGTGTTGGGCTTTT	qRT-PCR	This study
moaE-R1-qPCR / 2550	CGCCTTGATTGGTTTGTTCT	qRT-PCR	This study
F1-*torY*-qPCR / 2582	TGGGAAGGTTCTAGCCATTTT	qRT-PCR	This study
R1-*torY*-qPCR / 2583	CACACTCGTTGTGCCATTTC	qRT-PCR	This study
F1-*torZ*-qPCR / 2584	TCAAAGGTACTGTCGCTCTCC	qRT-PCR	This study
R1-*torZ*-qPCR / 2585	CGCCACCTGTTTAGGTTGTT	qRT-PCR	This study
moaA-F1-qPCR / 2614	ACCCGAAGCTAACAAACCAA	qRT-PCR	This study
moaA-R1-qPCR / 2615	TTTCTTCCAATCAGCCACATC	qRT-PCR	This study

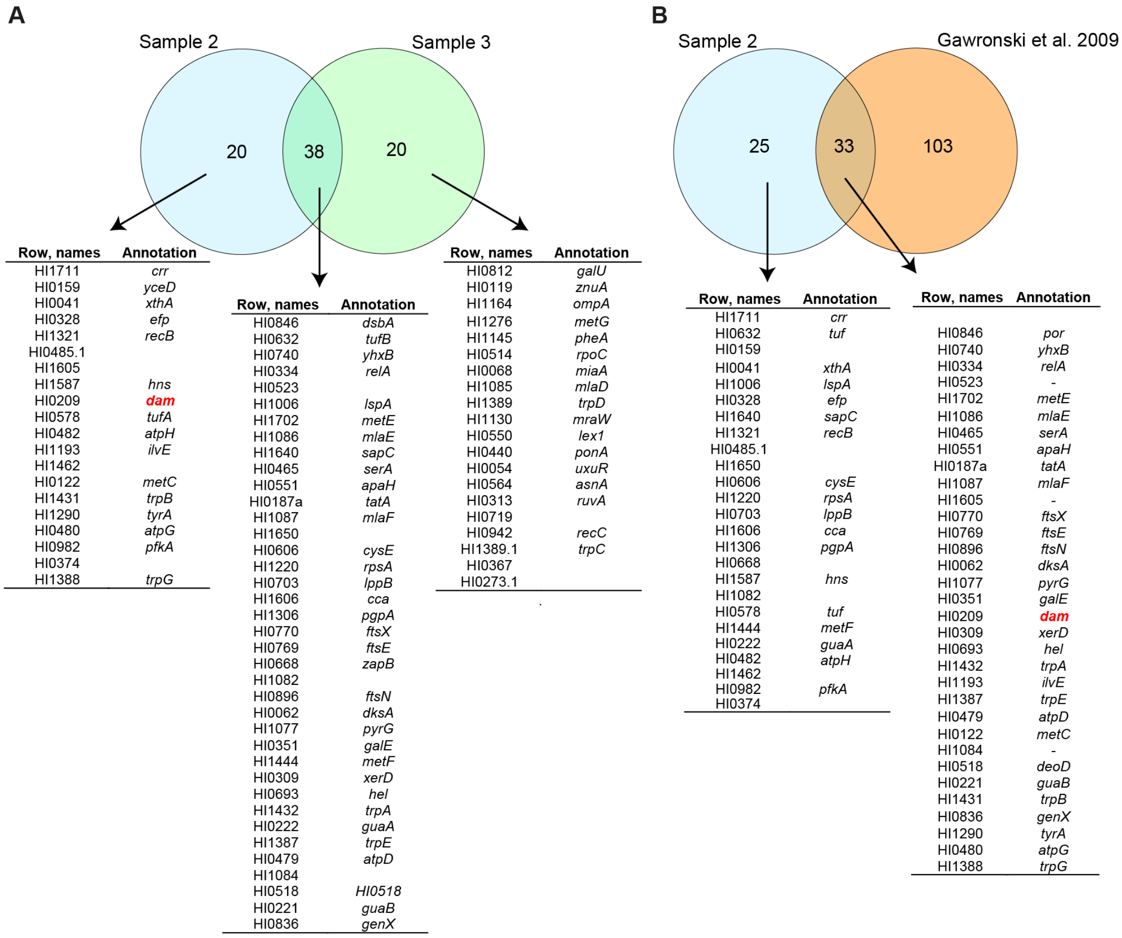

A – Sample 2 only (20)

Row, names	Annotation
HI1711	*crr*
HI0159	*yceD*
HI0041	*xthA*
HI0328	*efp*
HI1321	*recB*
HI0485.1	
HI1605	
HI1587	*hns*
HI0209	***dam***
HI0578	*tufA*
HI0482	*atpH*
HI1193	*ilvE*
HI1462	
HI0122	*metC*
HI1431	*trpB*
HI1290	*tyrA*
HI0480	*atpG*
HI0982	*pfkA*
HI0374	
HI1388	*trpG*

A – Common (38)

Row, names	Annotation
HI0846	*dsbA*
HI0632	*tufB*
HI0740	*yhxB*
HI0334	*relA*
HI0523	
HI1006	*lspA*
HI1702	*metE*
HI1086	*mlaE*
HI1640	*sapC*
HI0465	*serA*
HI0551	*apaH*
HI0187a	*tatA*
HI1087	*mlaF*
HI1650	
HI0606	*cysE*
HI1220	*rpsA*
HI0703	*lppB*
HI1606	*cca*
HI1306	*pgpA*
HI0770	*ftsX*
HI0769	*ftsE*
HI0668	*zapB*
HI1082	
HI0896	*ftsN*
HI0062	*dksA*
HI1077	*pyrG*
HI0351	*galE*
HI1444	*metF*
HI0309	*xerD*
HI0693	*hel*
HI1432	*trpA*
HI0222	*guaA*
HI1387	*trpE*
HI0479	*atpD*
HI1084	
HI0518	*HI0518*
HI0221	*guaB*
HI0836	*genX*

A – Sample 3 only (20)

Row, names	Annotation
HI0812	*galU*
HI0119	*znuA*
HI1164	*ompA*
HI1276	*metG*
HI1145	*pheA*
HI0514	*rpoC*
HI0068	*miaA*
HI1085	*mlaD*
HI1389	*trpD*
HI1130	*mraW*
HI0550	*lex1*
HI0440	*ponA*
HI0054	*uxuR*
HI0564	*asnA*
HI0313	*ruvA*
HI0719	
HI0942	*recC*
HI1389.1	*trpC*
HI0367	
HI0273.1	

B – Sample 2 only (25)

Row, names	Annotation
HI1711	*crr*
HI0632	*tuf*
HI0159	
HI0041	*xthA*
HI1006	*lspA*
HI0328	*efp*
HI1640	*sapC*
HI1321	*recB*
HI0485.1	
HI1650	
HI0606	*cysE*
HI1220	*rpsA*
HI0703	*lppB*
HI1606	*cca*
HI1306	*pgpA*
HI0668	
HI1587	*hns*
HI1082	
HI0578	*tuf*
HI1444	*metF*
HI0222	*guaA*
HI0482	*atpH*
HI1462	
HI0982	*pfkA*
HI0374	

B – Common (33)

Row, names	Annotation
HI0846	*por*
HI0740	*yhxB*
HI0334	*relA*
HI0523	-
HI1702	*metE*
HI1086	*mlaE*
HI0465	*serA*
HI0551	*apaH*
HI0187a	*tatA*
HI1087	*mlaF*
HI1605	-
HI0770	*ftsX*
HI0769	*ftsE*
HI0896	*ftsN*
HI0062	*dksA*
HI1077	*pyrG*
HI0351	*galE*
HI0209	***dam***
HI0309	*xerD*
HI0693	*hel*
HI1432	*trpA*
HI1193	*ilvE*
HI1387	*trpE*
HI0479	*atpD*
HI0122	*metC*
HI1084	-
HI0518	*deoD*
HI0221	*guaB*
HI1431	*trpB*
HI0836	*genX*
HI1290	*tyrA*
HI0480	*atpG*
HI1388	*trpG*

Figure S1. Summarized outcome of genome-wide screening of *H. influenzae* genes required for *in vivo* survival.

(A) Venn diagram summarizing analysis-specific and commonly found underrepresented genes when comparing *output* (sample 2 and 3) *versus input* (sample 1) libraries. Thirty eight genes were commonly underrepresented in both analyses. **(B)** Venn diagram shows the number of commonly underrepresented genes when comparing sample 2 (normal lung function, 24 hpi) with the previously published list of genes generated by HITS, using normal lung function mice and 24 hpi (15). Thirty three genes were commonly underrepresented in both analyses, one of them is the *dam* gene, indicated in red in both panels A and B.

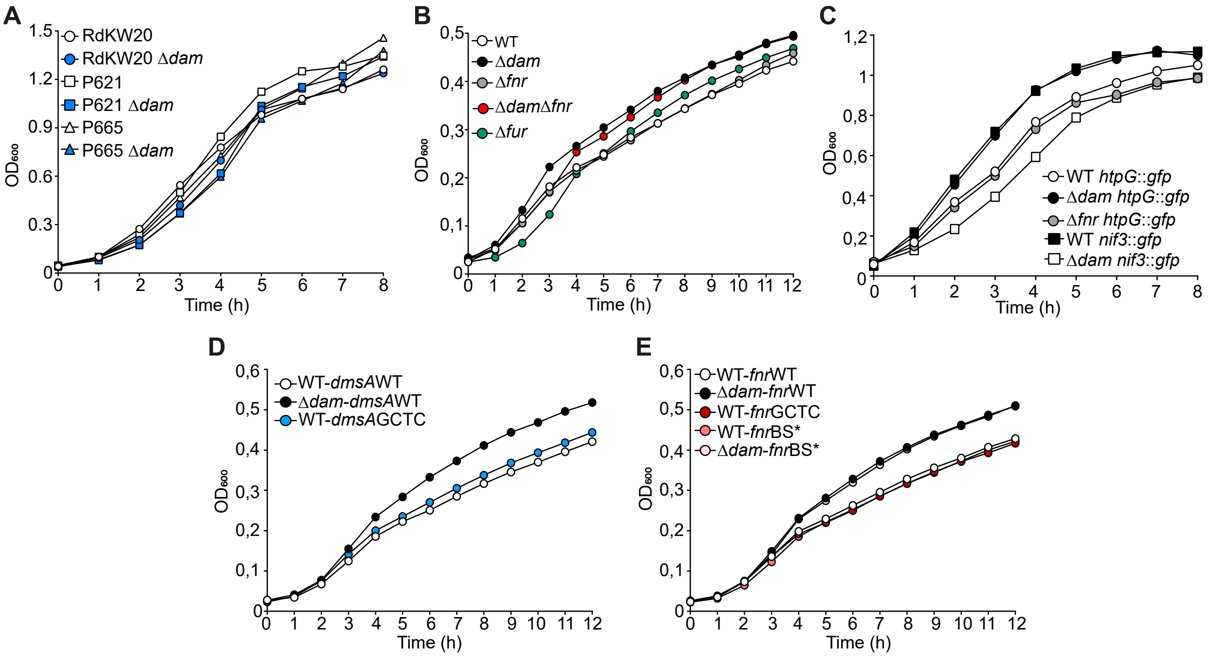

Figure S2. Growth of *H. influenzae* strains used in this study.
(A) Growth of WT and Δ*dam* strains in sBHI at 37ºC with 5% CO_2, in 25 mL sBHI with shaking; OD_{600} was recorded every hour for 8 h. Statistical analyses showed significant differences as follows: the RdKW20Δ*dam* strain showed higher OD_{600} than the RdKW20WT from 1 to 8 h, $p<0.0001$; the P621Δ*dam* strain showed higher OD_{600} than the P621WT from 1 to 8 h; the P665Δ*dam* strain showed higher OD_{600} than the P665WT from 1 to 8 h. **(B)** Growth of RdKW20WT, Δ*dam*, Δ*fnr*, Δ*dam*Δ*fnr* and Δ*fur* strains in 96-well plates, OD_{600} was recorded every 1 h for 12 h. The Δ*dam* strain showed higher OD_{600} than the WT at 3 h, $p<0.01$; 4 h, $p<0.001$; from 5 to 12 h, $p<0.0001$; the Δ*dam*Δ*fnr* strain showed higher OD_{600} than the WT at 4 h, $p<0.05$; from 5 to 6 h, $p<0.01$; from 7 to 12 h, $p<0.0001$. **(C)** Growth of RdKW20 WT, Δ*dam* and Δ*fnr* strains carrying *htpG::gfp* and *nif3::gfp* transcriptional fusions. Strains were grown in sterile 250 mL flasks with 25 mL sBHI and shaking (180 r.p.m.); OD_{600} was recorded every hour for 8 h. Statistical analyses showed significant differences as follows: the Δ*dam-htpG::gfp* strain showed higher OD_{600} than WT-*htpG::gfp* (at 1 h, $p<0.001$; from 2 to 8 h, $p<0.0001$); Δ*fnr-htpG::gfp*, lower OD_{600} than WT-*htpG::gfp* (from 1 to 8 h, $p<0.0001$); Δ*dam-nif3::gfp*, lower OD_{600} than WT-*nif3::gfp* (from 1 to 8 h, $p<0.0001$). **(D)** Growth of RdKW20 WT-*dmsA*-WT, Δ*dam-dmsA*-WT and WT-*dmsA*-GCTC in 96-well plates; OD_{600} was recorded every 1 h for 12 h. Statistical analyses showed significant differences as it follows: RdKW20Δ*dam-dmsA*-WT, higher OD_{600} than WT-*dmsA*-WT (from 3 to 12 h, $p<0.0001$), and than WT-*dmsA*-GCTC (at 3 h, $p<0.01$; from 4 to 12 h, $p<0.0001$). **(E)** Growth of RdKW20WT-*fnr*WT, Δ*dam-fnr*-WT, WT-*fnr*-GCTC, WT-*fnr*BS* and Δ*dam-fnr*BS* in 96-well plates; OD_{600} was recorded every 1 h for 12 h. Statistical analyses showed significant differences as follows: RdKW20WT-*fnr*-WT *versus* WT-*fnr*-GCTC, at 11 h, $p<0.05$. In all cases, statistical comparisons of the means were carried out with a two-way ANOVA (Dunnett's multiple comparisons test and Tukey's multiple comparisons test).

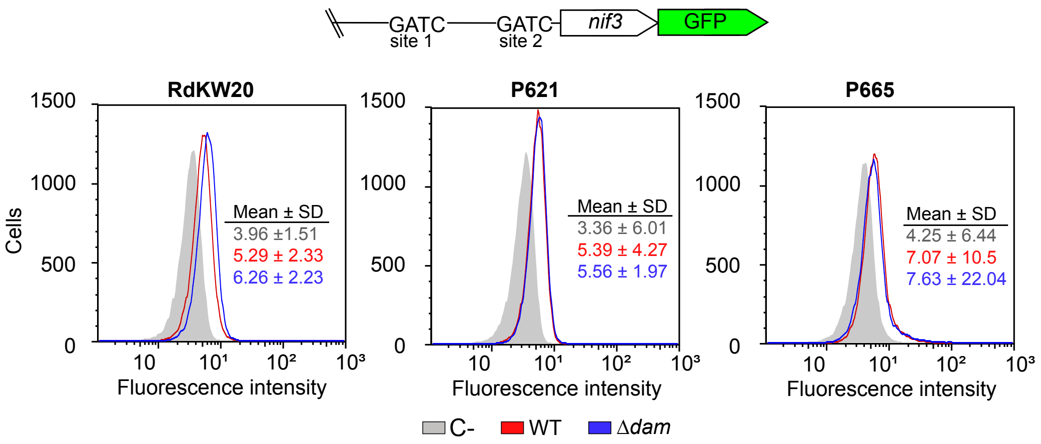

Figure S3. Flow cytometry analysis of *nif3* gene expression.
Flow cytometry analysis of *nif3* gene expression during aerobic mid-exponential bacterial growth. RdKW20 WT and Δ*dam* strains carrying a chromosomal *nif3-gfp* transcriptional fusion were used.

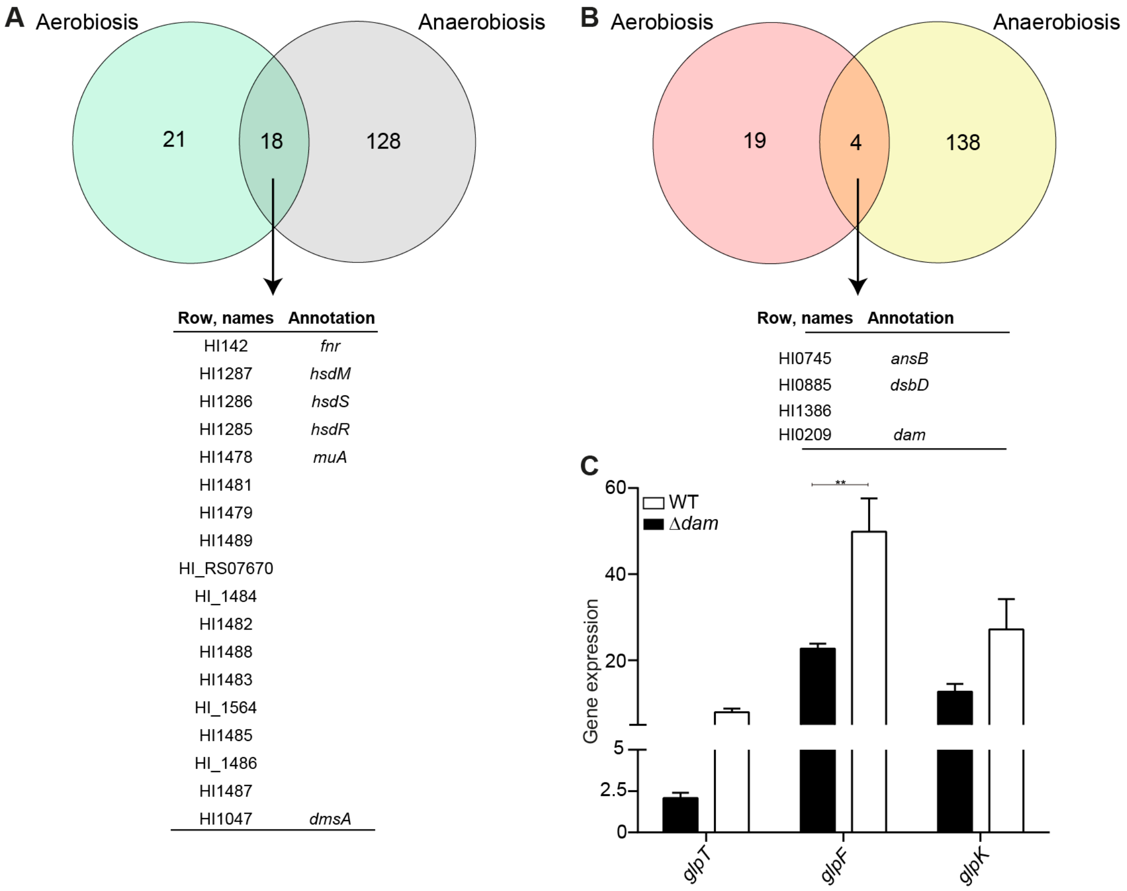

Figure S4. DEGs commonly found between assay conditions, aerobiosis and anaerobiosis. Venn diagrams show differentially expressed genes commonly found between RdKW20 WT and Δ*dam* strains grown in aerobiosis and anaerobiosis, **(A)** genes commonly upregulated upon *dam* inactivation (18); **(B)** genes commonly downregulated upon *dam* inactivation (4). **(C)** Expression of the *glpT, glpF* and *glpK* genes, under anaerobic condition, determined by RT-qPCR. Significant differences determined with two-way ANOVA (Tukey's multiple comparison test) (**, P<0.01).

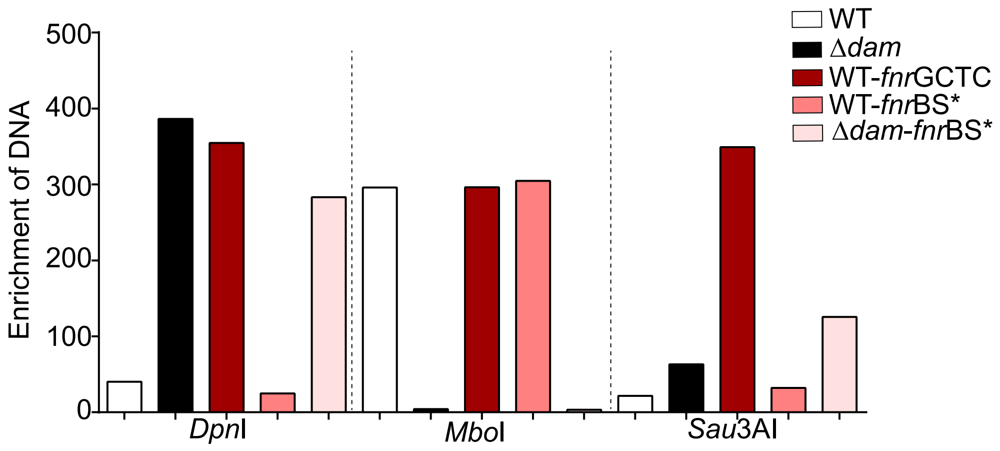

Figure S5. Analysis of GATC methylation in the promoter region of the *fnr* gene. Strains RdKW20 WT-*fnr*WT, Δ*dam*-*fnr*WT, WT-*fnr*GCTC, WT-*fnr*BS* and Δ*dam*-*fnr*BS* were tested. Cultures were grown in aerobiosis. qPCR using digested samples as templates confirmed lack of methylation in the RdKW20 WT-*fnr*GCTC.

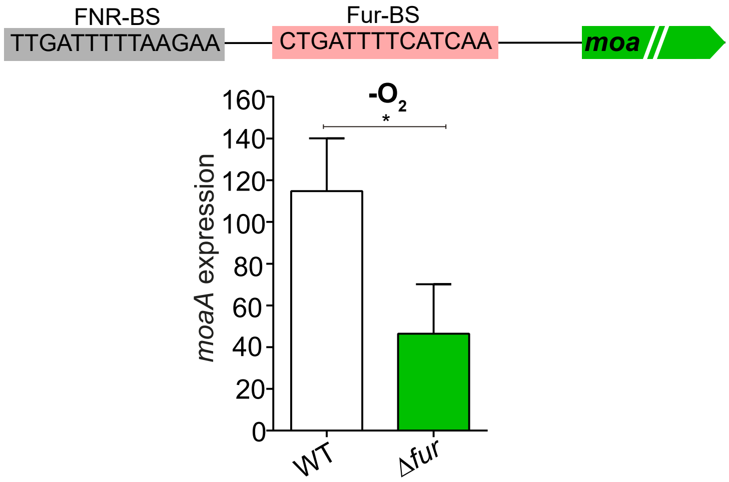

Figure S6. Expression of the *moaA* gene in RdKW20 WT and Δ*fur* strains by RT-qPCR. Strains were grown in anaerobiosis. Statistically significant differences were determined by t-test (*, $P<0.05$). Predicted FNR binding site is shown in grey; predicted Fur binding site is shown in pink.

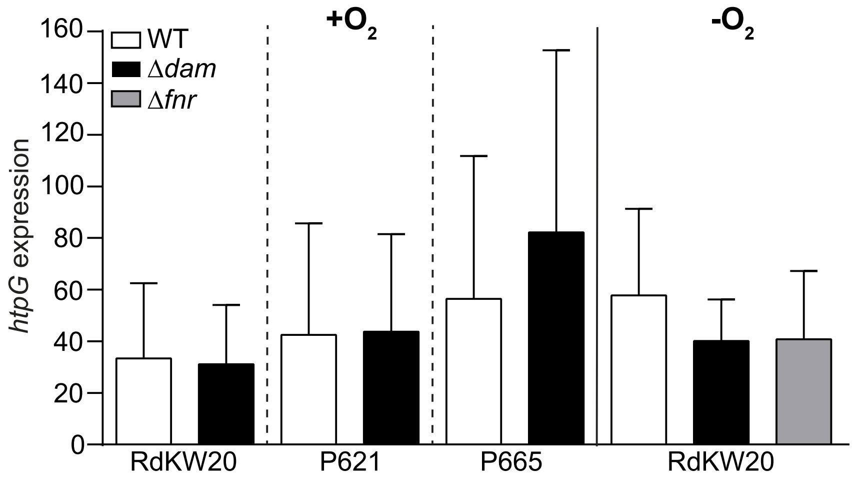

Figure S7. Expression of the *htpG* gene by RT-qPCR.

Total RNA was extracted from bacterial cultures grown in aerobiosis or anaerobiosis, and expression of the *htpG* gene was measured by RT-qPCR. Statistical analysis was performed by analysis of variance with two-way ANOVA (Tukey's multiple comparison test). Significant differences were not found.

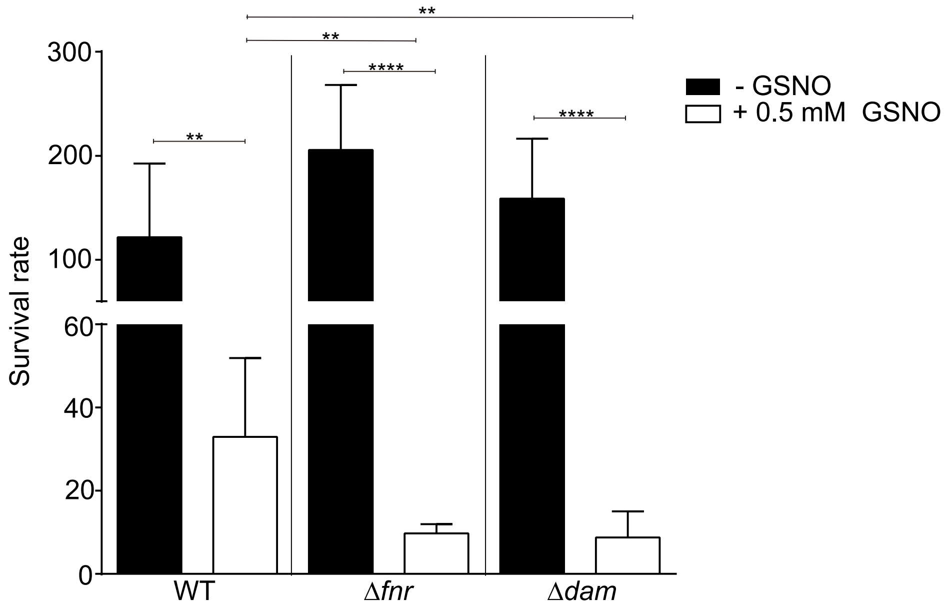

Figure S8. Survival of *H. influenzae* RdKW20 strains in the presence of the NO donor GNSO.
H. influenzae strains were incubated in sBHI containing 0.5 mM GSNO for 12 h and viability was assessed by CFU determination. Statistically significant differences were determined by two-way ANOVA (Tukey's multiple comparison test) (**, P<0.01; ****, P<0.0001).

References

1. Langereis J.D., Zomer A., Stunnenberg H.G., Burghout P. and Hermans, P.W.M. (2013) Nontypeable *Haemophilus influenzae* carbonic anhydrase is important for environmental and intracellular survival. *J. Bacteriol.*, **195**, 2737–2746.
2. Zomer, A., Burghout, P., Bootsma, H.J., Hermans, P.W.M. and van Hijum, S.A.F.T. (2012) Essentials: Software for rapid analysis of high throughput transposon insertion sequencing data. *PLoS One*, **7**, 1–9.
3. Dobin, A., Davis, C.A., Schlesinger, F., Drenkow, J., Zaleski, C., Jha, S., Batut, P., Chaisson, M. and Gingeras, T.R. (2013) STAR: ultrafast universal RNA-seq aligner. *Bioinformatics*, **29**, 15–21.
4. Putri, G.H., Anders, S., Pyl, P.T., Pimanda, J.E. and Zanini,F. (2022) Analysing high-throughput sequencing data in Python with HTSeq 2.0. *Bioinformatics*, **38**, 2943–2945.
5. Love, M.I., Huber, W. and Anders, S. (2014) Moderated estimation of fold change and dispersion for RNA-seq data with DESeq2. *Genome Biol.*, **15**, 550.
6. Ramírez, F., Ryan, D.P., Grüning, B., Bhardwaj, V., Kilpert, F., Richter, A.S., Heyne, S., Dündar, F. and Manke, T. (2016) deepTools2: a next generation web server for deep-sequencing data analysis. *Nucleic Acids Res.*, **44**, W160-5.
7. Shannon, P., Markiel, A., Ozier, O., Baliga, N.S., Wang, J.T., Ramage, D., Amin, N., Schwikowski, B. and Ideker, T. (2003) Cytoscape: a software environment for integrated models of biomolecular interaction networks. *Genome Res.*, **13**, 2498–2504.
8. Szklarczyk, D., Gable, A.L., Lyon, D., Junge, A., Wyder, S., Huerta-Cepas, J., Simonovic, M., Doncheva, N.T., Morris, J.H., Bork, P., *et al.* (2019) STRING v11: protein-protein association networks with increased coverage, supporting functional discovery in genome-wide experimental datasets. *Nucleic Acids Res.*, **47**, D607–D613.

9. Tracy, E., Ye, F., Baker, B.D. and Munson, R.S.J. (2008) Construction of non-polar mutants in *Haemophilus influenzae* using FLP recombinase technology. *BMC Mol. Biol.*, **9**, 101.
10. Fleischmann, R.D., Adams, M.D., White, O., Clayton, R.A., Kirkness, E.F., Kerlavage, A.R., Bult, C.J., Tomb, J.F., Dougherty, B.A., Merrick, J.M., *et al.* (1995) Whole-genome random sequencing and assembly of *Haemophilus influenzae* Rd. *Science (80-.).*, **269**, 496–512.
11. Moleres, J., Fernández-Calvet, A., Ehrlich, R.L., Martí, S., Pérez-Regidor, L., Euba, B., Rodríguez-Arce, I., Balashov, S., Cuevas, E., Liñares, J., *et al.* (2018) Antagonistic pleiotropy in the bifunctional surface protein FadL (OmpP1) during adaptation of Haemophilus influenzae to chronic lung infection associated with chronic obstructive pulmonary disease. *MBio*, **9**, 1–23.
12. Allen, S., Zaleski, A., Johnston, J.W., Gibson, B.W. and Apicella, M.A. (2005) Novel sialic acid transporter of *Haemophilus influenzae*. *Infect Immun*, **73**, 5291–5300.
13. Martin B , Prudhomme M, Alloing G, Granadel C, Claverys JP (2000) Cross-regulation of competence pheromone production and export in the early control of transformation in *Streptococcus pneumoniae*. Mol Microbiol, 38(4):867-78.
14. Burghout, P., Zomer, A., van der Gaast-de Jongh, C.E., Janssen-Megens, E.M., Françoijs, K.-J., Stunnenberg, H.G. and Hermans, P.W.M. (2013) *Streptococcus pneumoniae* folate biosynthesis responds to environmental CO_2 levels. *J. Bacteriol.*, **195**, 1573–1582.
15. Gawronski, J.D., Wong, S.M.S., Giannoukos, G., Ward, D. V. and Akerley, B.J. (2009) Tracking insertion mutants within libraries by deep sequencing and a genome-wide screen for *Haemophilus* genes required in the lung. *Proc. Natl. Acad. Sci. U. S. A.*, **106**, 16422–16427.
